# *N*-Acetylglucosamine and Immunoglobulin Strengthen Gut Barrier Integrity via Complementary Microbiome Modulation

**DOI:** 10.3390/nu18020210

**Published:** 2026-01-09

**Authors:** Emma De Beul, Jasmine Heyse, Michael Jurgelewicz, Aurélien Baudot, Lam Dai Vu, Pieter Van den Abbeele

**Affiliations:** 1 Cryptobiotix SA, Technologiepark-Zwijnaarde 82, 9052 Ghent, Belgium; emma.debeul@cryptobiotix.com (E.D.B.); jasmine.heyse@cryptobiotix.com (J.H.); aurelien.baudot@cryptobiotix.com (A.B.); lamdai.vu@cryptobiotix.com (L.D.V.); 2 Designs for Health Inc., 14 Commerce Blvd., Palm Coast, FL 32164, USA; mjurgelewicz@designsforhealth.com; 3 Human Nutrition Institute, University of Bridgeport, Bridgeport, CT 06604, USA

**Keywords:** serum-derived bovine immunoglobulin (SBI), *N*-acetylglucosamine (NAG), systemic intestinal fermentation research (SIFR^®^), gut barrier integrity, gut microbiome composition, short-chain fatty acids (SCFA), metabolomics

## Abstract

**Background**: Gut barrier dysfunction and altered gut microbial metabolism are emerging signatures of chronic gut disorders. Considering growing interest in combining structurally and mechanistically distinct bioactives, we investigated the individual and combined effects of serum-derived bovine immunoglobulin (SBI) and *N*-acetylglucosamine (NAG) on the gut microbiome and barrier integrity. **Methods**: The validated ex vivo SIFR^®^ (Systemic Intestinal Fermentation Research) technology, using microbiota from healthy adults (*n* = 6), was combined with a co-culture of epithelial/immune (Caco-2/THP-1) cells. **Results**: While SBI and NAG already significantly improved gut barrier integrity (TEER, transepithelial electrical resistance, +21% and +29%, respectively), the strongest effect was observed for SBI_NAG (+36%). This potent combined effect related to the observation that SBI and NAG each induced distinct, complementary shifts in microbial composition and metabolite output. SBI most selectively increased propionate (~Bacteroidota families) and health-associated indole derivatives (e.g., indole-3-propionic acid), while NAG most specifically boosted acetate and butyrate (~*Bifidobacteriaceae*, *Ruminococcaceae*, and *Lachnospiraceae*). The combination of SBI_NAG displayed effects of the individual ingredients, thus, for instance, enhancing all three short-chain fatty acids (SCFA) and elevating microbial diversity (CMS, community modulation score). **Conclusions**: Overall, SBI and NAG exert complementary, metabolically balanced effects on the gut microbiota, supporting combined use, particularly in individuals with gut barrier impairment or dysbiosis linked to lifestyle or early-stage gastrointestinal disorders.

## 1. Introduction

The gut microbiome is composed of trillions of microorganisms that play a critical role in human health, influencing digestion, nutrient absorption, immune function, and gut barrier integrity [1,2]. Disruption of this microbial ecosystem can contribute to the development and progression of a wide range of chronic conditions. Particularly, impaired gut barrier function, colloquially termed ‘leaky gut’, is recognized as a central issue in various diseases. Increased intestinal permeability allows translocation of toxins and microbes, triggering inflammation and systemic disease [3,4,5]. It has been implicated in gastrointestinal disorders (inflammatory bowel disease (IBD) [6], celiac disease [7], and irritable bowel syndrome (IBS) [8]), metabolic conditions (diabetes [9], obesity, and non-alcoholic fatty liver disease [10]), auto-immune diseases (lupus [11], ankylosing spondylitis [12], and psoriasis [13]), allergic responses (allergies [14], asthma [15], and eczema [16]), and neurological or psychiatric conditions (autism [17], ADHD [18], depression [19], and dementia [20]). Given the pivotal role of the gut barrier and the influence of the gut microbiome on its integrity, there is growing interest in microbiome-targeted interventions that restore or strengthen gut barrier integrity.

Two such interventions include serum-derived bovine immunoglobulin (SBI) and *N*-acetylglucosamine (NAG). SBI has been shown to enhance gut barrier integrity and reduce inflammation by binding microbial antigens and in doing so, prevent translocation [21,22,23,24,25,26]. In addition, SBI modulates the gut microbiota [21,24,25,27,28,29], thereby promoting the production of beneficial metabolites like short-chain fatty acids (SCFA) and indole-3-propionic acid [27,29], which contribute to epithelial health. In a clinical study involving HIV patients with chronic diarrhea under suppressive antiretroviral therapy, SBI supplementation reduced systemic inflammation and improved gut integrity [30]. Additional studies have reported symptom relief by SBI in conditions such as IBD [31,32], IBS [33,34,35], HIV-associated enteropathy [36], and COVID-19 [37]. In addition, unlike the protein-based SBI, NAG is a monosaccharide commonly found as component of mucin glycoproteins, which are essential for forming a dense, protective mucus layer that shields the epithelium from bacterial invasion [38,39]. A preclinical study (mouse models of ulcerative colitis) demonstrated that NAG enhanced epithelial repair, lowered intestinal permeability, reduced inflammation and suppressed the overgrowth of pathogenic bacteria [40]. Similar restorative effects on the mucus barrier were observed in a rat model of diarrhea-predominant IBS [41]. Early clinical findings from a pilot study in children with treatment-resistant IBD suggest that oral NAG may alleviate symptoms and improve epithelial morphology [42].

While both SBI and NAG have shown promise individually in improving gut barrier integrity, their combined use has not yet been investigated. However, their different modes of action, i.e., SBI as protein-based antigen binder and NAG as carbohydrate-based mucus support, make it plausible that their effects on gut barrier and microbiome could complement each other. This study aimed to explore whether combining SBI and NAG would enhance the overall profile compared to each ingredient alone, or whether antagonistic interactions might diminish efficacy. Understanding this interplay is essential for guiding development of multi-component strategies to support gut health.

The ex vivo SIFR^®^ technology (Systemic Intestinal Fermentation Research) is well suited to address this knowledge gap as it enables studying the human gut microbiota and host interactions under physiologically relevant conditions. Owing to its high throughput, it enables capturing interindividual variability across test subjects. Most importantly, it provides predictive insights into clinical outcomes, including microbiome modulation and gut barrier function [27,43].

The present study aimed to elucidate the individual and combined effects of SBI and NAG on the human adult gut microbiome and how this may impact gut barrier integrity. Using the ex vivo SIFR^®^ technology, we demonstrated that SBI and NAG exerted distinct yet complementary effects on the microbiome and doing so promoted barrier integrity, with their combination delivering the most pronounced and balanced impact. This highlights the potential of combining structurally distinct bioactives to support gut health.

## 2. Materials and Methods

### 2.1. Test Compounds

Compounds were evaluated as follows: serum-derived bovine immunoglobulin (SBI, commercial name: Immunolin, Proliant Health & Biologicals, Des Moines, IA, USA), *N*-acetylglucosamine (NAG, Designs for Health, Palm Coast, FL, USA), and their combination (SBI_NAG, commercial name: IgGI Shield^TM^, Designs for Health, Palm Coast, FL, USA). SBI was tested at a dose equivalent to 2.5 g/day, while NAG was tested at 1 g/day (corresponding to ex vivo test concentrations of 2.5 and 1 g/L). The test products used in this study were commercially available and were analytically characterized prior to experimentation. Manufacturer Certificates of Analysis confirmed identity, composition, purity, and microbiological safety for both ingredients. Test products were compared with a no-substrate control (NSC), where the colonic fermentation was simulated without the addition of test products.

### 2.2. Simulation of Upper Gastrointestinal Tract Digestion

Oral, gastric, and small intestinal digestion and absorption of SBI in the upper gastrointestinal tract was simulated largely according to the INFOGEST 2.0 method [44]. The necessary adjustments were made to ensure compatibility with simulation of the colonic fermentation, including oxygen removal and mimicking small-intestinal absorption using dialysis membranes, as described earlier [29]. For NSC and NAG, no digestion was performed. Water was used for these study arms during digestion and NAG was added post-digestion because its small molecular size would result in near-complete removal during the dialysis step, preventing meaningful evaluation of its colonic effects.

### 2.3. Fecal Microbiota Sourcing

To simulate biorelevant gut microbiota ex vivo using the SIFR^®^ technology (Cryptobiotix, Ghent, Belgium), fresh fecal samples were obtained from six human adults (mean age: 40.7 ± 10.1 years; test subjects 1 (male, 48), 2 (female, 49), 3 (male, 35), 4 (female, 52), 5 (female, 30), and 6 (female, 31)). Participants had not used antibiotics within three months before donation and had no history of gastrointestinal disorders (cancer, ulcers, and inflammatory bowel disease). The study received approval from the Ethics Committee of the University Hospital Ghent (reference number BC-09977; approval date: 13 April 2021). All test subjects provided written informed consent for anonymous use of their fecal samples and scientific publication of anonymized study results. The fecal samples were kept under anaerobic conditions upon collection to minimize the effect of storage and transport on the microbiota.

### 2.4. Ex Vivo Simulation of Colonic Fermentation via SIFR^®^ Technology

Ex vivo SIFR^®^ experiments were performed as outlined in recent work [43]. Briefly, individual fecal samples were processed in a bioreactor management device (Cryptobiotix, Ghent, Belgium). Each bioreactor was filled with 5 mL containing a nutritional medium (M0017, Cryptobiotix, Ghent, Belgium), a freshly collected fecal inoculum from a single test subject, and the appropriate test product(s), after which the bioreactors were individually sealed and rendered anaerobic. They were subsequently incubated under continuous agitation (140 rpm) at 37 °C for 24 h (MaxQ 6000, Thermo Scientific, Merelbeke, Belgium).

**Figure 1:**
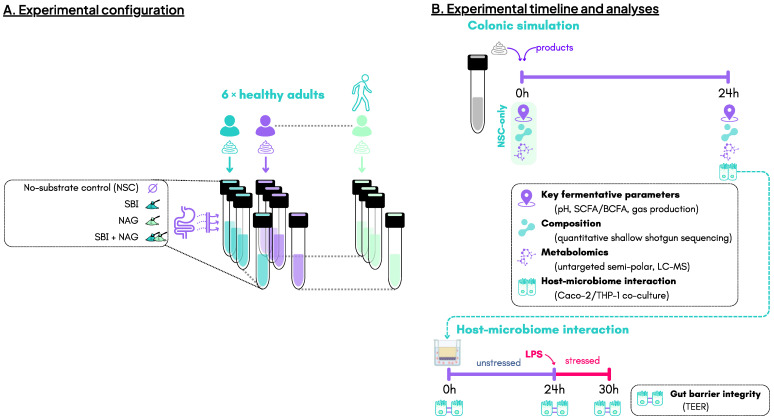
**The impact of serum-derived bovine immunoglobulin (SBI) and *N*-acetylglucosamine (NAG) on the gut microbiota of healthy adults (*n* = 6) was assessed using the ex vivo SIFR^®^ technology.** (**A**) Experimental configuration, and (**B**) timeline and analysis. BCFA: branched-chain fatty acids, LC-MS: liquid chromatography–mass spectrometry; LPS: lipopolysaccharide, NSC: no-substrate control; SCFA: short-chain fatty acids, SIFR^®^: systemic intestinal fermentation research; TEER: transepithelial electrical resistance.

Four study arms were tested for each test subject (*n* = 6): (i) no-substrate control (NSC), (ii) SBI, (iii) NAG, and (iv) the combination of SBI and NAG (SBI_NAG) (Figure 1A). At 0 h (NSC only) and after 24 h of incubation, headspace gas pressure was measured and liquid samples were collected to analyze key fermentative parameters, microbial composition, metabolic changes, and host–microbiome interactions (Figure 1B).

Treatment effects of SBI, NAG, and SBI_NAG were evaluated by comparing with NSC at 24 h (parallel unsupplemented control where the microbial inoculum was grown under identical conditions but without test products). Two additional technical replicates were run only in the NSC study arm for each test subject to confirm the high technical reproducibility of the SIFR^®^ technology [43]. Indeed, coefficients of variation for pH, gas, and SCFA (acetate, propionate, and butyrate) measurements were below 3% for the NSC technical replicates.

### 2.5. Key Fermentation Parameters

Short-chain fatty acids (SCFA; acetate, propionate, butyrate, and valerate) and branched-chain fatty acids (BCFA; sum of isobutyrate, isovalerate, and isocaproate) were extracted using diethyl ether and quantified using gas chromatography with flame ionization detection, as previously described [45]. Briefly, 0.5 mL samples were diluted in distilled water (1:3), acidified with 0.5 mL of 48% sulfuric acid, and supplemented with an excess of sodium chloride, 0.2 mL of internal standard (2-methylhexanoic acid), and 2 mL of diethyl ether. After homogenization and phase separation, the diethyl ether layer was collected and analyzed using a Trace 1300 chromatograph (Thermo Fisher Scientific, Merelbeke, Belgium) equipped with a Stabilwax-DA capillary GC column, a flame ionization detector, and a split injector using nitrogen gas as the carrier and makeup gas. The injection volume was 1 µL and the temperature ranged from 110 °C to 240 °C. The temperatures of the injector and detector were 240 and 250 °C, respectively. pH was measured using an electrode (Hannah Instruments Edge HI2002, Temse, Belgium).

### 2.6. Untargeted Metabolite Profiling

Liquid chromatography–mass spectrometry (LC-MS) analysis was conducted using a Thermo Scientific Vanquish LC coupled to a Thermo Q Exactive HF MS (Thermo Scientific), equipped with an electrospray ionization source. The analysis was performed in both positive and negative ionization mode. The UPLC protocol was based on the method described by Doneanu et al. [46], with slight modification. Peak areas were extracted using Compound Discoverer 3.1 (Thermo Scientific), complemented by manual extraction based on an in-house library using Skyline 21.1 (MacCoss Lab Software, Seattle, WA, USA) [47]. Compound identification was performed at different levels, i.e., level 1 (retention times compared against in-house authentic standards, accurate mass with an accepted deviation of 3 ppm, and MS/MS spectra), level 2a (retention times and accurate mass), level 2b (accurate mass and MS/MS spectra), and level 3 (accurate mass alone). The analysis focused on level 1 and 2a metabolites (to ensure correct annotation) that increased in at least 4 test subjects for one or more treatments between 0 h and 24 h, suggesting their production by gut microbes. Technical reproducibility was assessed by including a QC sample (pooled sample of all samples) every six injections. These QC samples clustered together in the exploratory analysis of level 1-annotated metabolites, confirming the high technical reproducibility of the method, with an average coefficient of variation of 8.1%.

### 2.7. Taxonomic Microbiota Analysis by Quantitative Shallow Shotgun Sequencing

Quantitative insights into microbial composition were obtained by multiplying relative abundances (%; shallow shotgun sequencing) with total cell count (cells/mL; flow cytometry) to obtain estimated cell densities (cells/mL) for each taxonomic level (phylum, family, genus, and species) in each sample.

DNA was extracted via the SPINeasy DNA Kit for Soil (MP Biomedicals, Eschwege, Germany), according to manufacturer’s instructions. Subsequently, libraries were prepared using the Watchmaker DNA Library Prep Kit, with genomic DNA being fragmented using a master mix of Watchmaker Frag/AT Buffer and Frag/AT Enzyme Mix (Watchmaker Genomics, Boulder, CO, USA). IDT xGen UDI Primers and IDT Stubby Adapters were added and DNA libraries were constructed through 7 cycles of PCR, after which they were purified utilizing CleanNGS magnetic beads (CleanNA, Waddinxveen, The Netherlands) and eluted into nuclease-free water. Subsequently, library quantification was conducted using the Qubit™ Fluorometer dsDNA HS Assay Kit (Thermo Fisher Scientific, Waltham, MA, USA). Libraries were circularized following the Element Adept library compatibility workflow and then sequenced on the Element AVITI platform using the AVITI 2 × 150 Cloudbreak sequencing kit (Element Biosciences, San Diego, CA, USA). The CosmosID-HUB Microbiome Platform was used to convert unassembled sequencing reads to relative abundances (%) of taxa [48,49] (https://app.cosmosid.com/, CosmosID Inc., Germantown, MD, USA; accessed on 19 June 2025). This platform was also used to calculate the Chao1 and Simson diversity index.

To determine total cell counts, samples were diluted in anaerobic phosphate-buffered saline (PBS), followed by cell staining with SYTO 16 at a final concentration of 1 µM, and counted via a BD FACS Verse flow cytometer (BD, Erembodegem, Belgium). Data were analyzed using FlowJo, version 10.8.1.

### 2.8. Host–Microbiome Interaction Assay

To understand how the modulation of the gut microbiome by the test products (as assessed via the SIFR^®^ technology) affected host cells, a coculture model was used, as previously described [27]. The model consisted of human epithelial cells (human adenocarcinoma Caco-2 cell line, ATCC^®^ HTB-37™) and immune cells (human acute monocytic leukemia THP1 cell line, differentiated to activated macrophages by a PMA treatment, ATCC^®^ TIB-202™), both obtained from the American Type Culture Collection (ATCC, Manassas, VA, USA). After 14 days of differentiation for Caco-2 cells and 2 days for THP-1 cells, immune cells were overlaid with an epithelial layer in a permeable well insert to simulate the gut wall. Colonic samples derived from the 24 h SIFR^®^-incubation were centrifuged and filter-sterilized (pore size: 0.22 μm) before apical administration in the coculture model as 20% of the cell medium (MEM medium supplemented with 1× NEAA and 1 mM sodium pyruvate with 10% FBS, Gibco, Carlsbad, CA, USA). This approach enables testing the interactions between the cells and metabolites in the colonic samples, without involving the microbes. The host–microbiome interaction assay consisted of two phases: (i) 24 h treatment with colonic samples to evaluate gut barrier integrity under unstressed conditions, and (ii) an additional 6 h incubation with 500 ng/mL lipopolysaccharide (Ultrapure LPS from *E. coli* 0111:B4, category number: tlrl-3pelps, Invitrogen, Carlsbad, CA, USA) to evaluate effects under stressed conditions. Gut barrier integrity was assessed by measuring transepithelial electrical resistance (TEER) [50] at 0 h, 24 h (before LPS addition), and 30 h (after LPS treatment). All samples were run in duplicate for quality control. Additional control conditions included two blanks (medium only, with or without LPS added after 24 h) and two positive controls with the anti-inflammatory compounds dexamethasone (D) or hydrocortisone (HC), where LPS was added after 24 h.

### 2.9. Data Analysis

Data analysis was performed using R (version 4.4.3; www.r-project.org; accessed on 8 September 2025). The ggplot2 package (v4.0.0) was used to make a series of violin plots, bar charts, and heat maps. For principal component analysis (PCA), data was scaled to unit variance and analyzed using the FactoMineR package v2.12 [51]. The two additional NSC technical replicates were not considered for statistical evaluation. Statistical analysis was conducted using a linear mixed-effects model, which accounted for both fixed effects (treatment) and random effects (interpersonal variation) (glmmTMB package v1.1.11 [52]). Effects of SBI, NAG, and SBI_NAG compared to the reference (NSC) were assessed via post hoc pairwise comparison using estimated marginal means (emmeans package v1.11.2). The Benjamini–Hochberg correction [53] was applied to control the false discovery rate (FDR) across parameters (stats package v3.6.2). For the statistical evaluation of the effects on microbial composition and metabolite production, the linear mixed-effects model was first applied to test the overall effect of treatments on families/species/metabolites, and only the families/species/metabolites that displayed significant overall treatment effects were further considered for post hoc pairwise comparison. For compositional analysis, statistical analysis was only performed on the abundance that has been corrected with the total cell counts (cells/mL) and not on the relative abundance (%). Missing values were dealt with as elaborated by Van den Abbeele et al. (2023) [43]. Effects were considered significant at *p*_adjusted_ < 0.05. In violin plots and bar charts, statistical differences between treatments and NSC are indicated with * (0.01 < *p*_adjusted_ < 0.05), ** (0.001 < *p*_adjusted_ < 0.01), or *** (*p*_adjusted_ < 0.001). In violin plots, significant differences between SBI and NAG or SBI_NAG are indicated with $/$$/$$$ and significant differences between NAG and SBI_NAG are indicated with &/&&/&&&. Additionally, ranks of average values per study arm are indicated above the horizontal axis. The community modulation score (CMS) [54] was calculated for both species and genus level, using the 150 most common species and corresponding 60 most common genera (covering on average 91.7% and 98.1%, of the microbial communities, respectively). The CMS measures the balance between the number of species/genera that are stimulated and those that are suppressed.

## 3. Results

### 3.1. Fecal Microbiota of Six Adults Captured Interpersonal Variation

**Figure 2:**
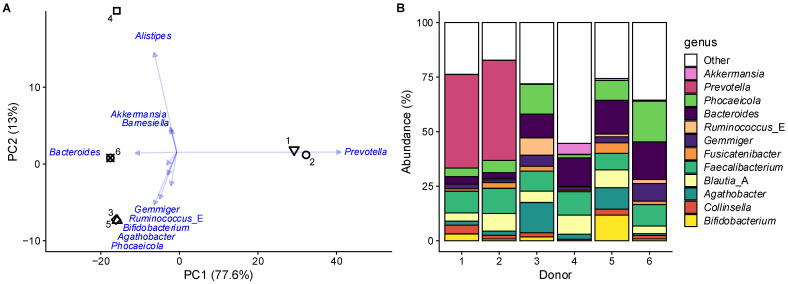
**The fecal microbiome of six healthy adults covered interpersonal differences**. (**A**) Principal component analysis (PCA) based on centered abundances at genus level (%) with indication of the individual fecal microbiota of the six test subjects (healthy human adults; unique symbols) along with loading vectors for the ten genera that explained most variation; (**B**) abundances (%) of the most abundant genera for each test subject (*n* = 1 per test subject).

Principal component analysis (PCA) at genus level (Figure 2A) revealed that most variation between the test subjects was captured along PC1 (77.6%), primarily related to high *Prevotella* abundance in subjects 1 and 2. The other subjects were distinguished by elevated levels of *Bacteroides* and *Phocaeicola* (Figure 2). In addition, subjects 3 and 5 exhibited high abundance of Bacillota_A genera (previously classified as Firmicutes) such as *Ruminococcus*_E, *Gemmiger*, *Fusicatenibacter*, *Faecalibacterium*, *Blautia*_A, and *Agathobacter* (Figure 2). The taxa mentioned above have been identified as key markers distinguishing different enterotypes within the human population [55,56]. Beyond enterotype-related genera, subject 4 displayed markedly higher *Alistipes* levels, while *Bifidobacterium* abundance was elevated in subject 5. These pronounced interindividual differences suggest that the microbiomes of the six test subjects in this study encompassed sufficient diversity to reflect the human adult population, ensuring representative findings.

### 3.2. SBI, NAG, and Especially Their Combination Enhanced Gut Barrier Integrity

**Figure 3:**
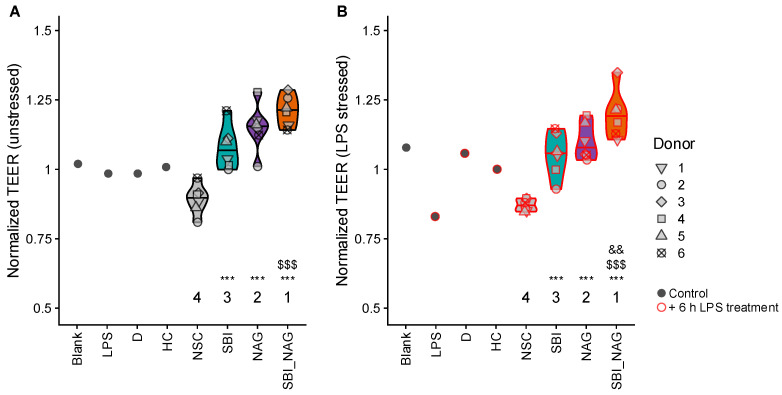
**SBI, NAG and SBI_NAG promoted gut barrier integrity**. Gut barrier integrity was measured as transepithelial electrical resistance (TEER), normalized to baseline (0 h). (**A**) Under unstressed conditions, measured after 24 h incubation with SIFR^®^-derived colonic samples of six test subjects; (**B**) under stressed conditions, measured after 30 h, following an additional 6 h treatment with lipopolysaccharide (LPS). Statistical differences between NSC and individual treatments are indicated with asterisk(s) (*), while differences between SBI and NAG or SBI_NAG are indicated with dollar sign(s) ($) and differences between NAG and SBI_NAG are indicated with ampersand(s) (&). The number of *, $ and & indicates the range of the adjusted *p*-value: one (0.01 < *p*_adjusted_ < 0.05), two (0.001 < *p*_adjusted_ < 0.01), or three symbols (*p*_adjusted_ < 0.001). The ranks of the average values per treatment are shown as well. Four control samples (*n* = 1) were also included in the visualization. Absolute TEER values are shown in Figure S1.

The upper gastrointestinal digestion and absorption and subsequent colonic fermentation of SBI, NAG, and SBI_NAG were simulated ex vivo using the SIFR^®^ technology. The colonic samples were applied to a Caco-2/THP-1 coculture model to assess how host–microbiome interactions affected gut barrier integrity. First, the control samples showed that a 6 h LPS exposure significantly reduced TEER compared to the blank without LPS treatment (Figure 3B). As expected, administering the anti-inflammatory compounds D and HC partially restored TEER values affected by LPS, bringing them closer to the value in the blank sample (Figure 3B).

After 24 h of interaction between colonic samples and the coculture, both SBI and NAG significantly increased TEER compared to NSC (+21% and +29%; *p*_adjusted_ = 0.0001 and 4 × 10^−6^, respectively), indicating a strengthened epithelial barrier (Figure 3A). This effect was even greater with the combined treatment (SBI_NAG) (+36%, *p*_adjusted_ = 4 × 10^−7^), which significantly outperformed SBI (*p*_adjusted_ = 0.003). These observations persisted under subsequent LPS-induced stress (Figure 3B). Again, the blend SBI_NAG showed the strongest effect (+37%), significantly exceeding both individual products (*p*_adjusted_ < 0.02).

### 3.3. Combining SBI and NAG Integrated Their Distinct Impacts on Gut Microbiota Composition

**Figure 4:**
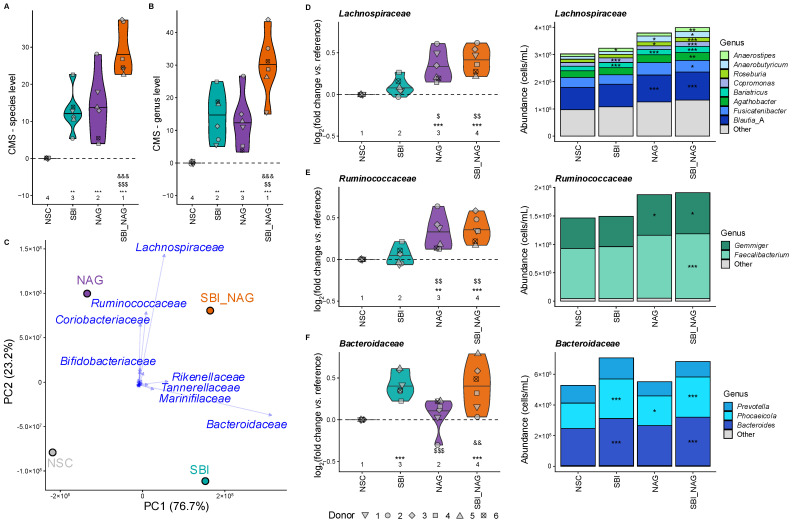
**SBI, NAG, and SBI_NAG specifically enhanced the growth of SCFA-producing taxa**. (**A**,**B**) Impact on microbial diversity, measured as the community modulation score (CMS) [54] at (**A**) species and (**B**) genus level for the six test subjects; (**C**) PCA summarizing treatment effects on family-level microbial composition based on centered estimated cell counts (cells/mL) as quantified via shallow shotgun sequencing and flow cytometry. Families that explained most variation are indicated in blue; (**D**–**F**) impact on key SCFA-producing families: (**D**) *Lachnospiraceae*, (**E**) *Ruminococcaceae*, and (**F**) *Bacteroidaceae*. Left: log_2_-transformed fold change relative to NSC for the six test subjects; right: estimated cell counts (cells/mL) of the most abundant genera, averaged across the six test subjects. Statistical differences between NSC and individual treatments are indicated with asterisk(s) (*), while differences between SBI and NAG or SBI_NAG are indicated with dollar sign(s) ($) and differences between NAG and SBI_NAG are indicated with ampersand(s) (&). The number of *, $ and & indicates the range of the adjusted *p*-value: one (0.01 < *p*_adjusted_ < 0.05), two (0.001 < *p*_adjusted_ < 0.01), or three symbols (*p*_adjusted_ < 0.001). The ranks of the average values per treatment are shown as well. For bar plots, only statistical differences relative to NSC are shown.

The gut microbiota composition upon treatment with SBI, NAG, and SBI_NAG was analyzed using quantitative shallow shotgun sequencing. All treatments enhanced microbial diversity, as quantified via the community modulation score (CMS) at both species and genus level (*p* < 0.006, Figure 4A,B). SBI_NAG outperformed both individual products, with CMS scores more than twice those observed for SBI or NAG alone (*p* < 0.002).

Next, the product-specific taxonomic shifts underlying these diversity changes were investigated. PCA indicated that both SBI and NAG strongly influenced the gut microbiota, with distinct specificity (Figure 4C and Figures S2–S4).

SBI shifted primarily to the right of NSC along PC1, reflecting increased abundances of propionogenic Bacteroidota families (*Bacteroidaceae*, *Rikenellaceae*, *Tannerellaceae*, *Marinifilaceae*) (Figure 4C and Figures S2–S4). Within the most prominent family, *Bacteroidaceae*, SBI specifically enriched *Bacteroides* spp. (most notably *B. uniformis*, *B. caccae*, *B. thetaiotaomicron*, *B. ovatus*, and *B. xylanisolvens*) and *Phocaeicola* spp. (*P. vulgatus* and *P. dorei*) (Figure 4F). Additional Bacteroidota that were promoted by SBI included *Alistipes* spp. (*Rikenellaceae*), *Parabacteroides* spp. (*Tannerellaceae*), *Odoribacter splanchnicus* and *Butyricimonas* spp. (*Marinifilaceae*), and *Barnesiella intestinihominis* (Barnesiellaceae). Beyond Bacteroidota, SBI also increased the propionate-producing families *Anaerotignaceae* (mainly *Anaerotignum faecicola*) and *Dialisteraceae* (mainly *Dialister* spp.) (Figures S2–S4). Finally, SBI also exerted a mild but significant stimulatory effect on several butyrate producers within Bacillota_A, including *Anaerostipes hadrus*, *Coprococcus*_A *catus*_A, *Bariatricus comes* (*Lachnospiraceae*), and *Agathobaculum butyriciproducens* (*Butyricicoccaceae*). Other *Lachnospiraceae* members were also stimulated, including *Copromonas* spp., *Dorea*(_A) spp. and *Enterocloster* spp. (Figure 4D and Figures S2–S4).

In contrast, NAG moved upwards along PC2 in the PCA (Figure 4C), indicating a high specificity of NAG for stimulating Bacillota_A (*Ruminococcaceae*, *Lachnospiraceae*) and Actinomycetota (*Bifidobacteriaceae* and *Coriobacteriaceae*) (Figure 4C and Figures S2–S4). Major butyrate producers enriched by NAG belonged to families *Lachnospiraceae* (*Anaerobutyricum hallii*, *Roseburia* spp., *Lachnospira eligens*, *Eubacterium*_G, *A. hadrus*, *C. catus*_A, *B. comes*, Figure 4D) and *Ruminococcaceae* (*Faecalibacterium* spp. and *Gemmiger* spp., Figure 4E). Additional *Lachnospiraceae* members promoted by NAG included different *Blautia* species, *Mediterraneibacter* spp. (*M. torques* and *M. faecis*) and *Oliverpabstia faecicola* (Figures S2–S4). Unlike SBI, NAG demonstrated strong bifidogenic effects, increasing the levels of acetate/lactate-producing *Bifidobacteriaceae* (mainly *Bifidobacterium adolescentis*). In the same Actinomycetota phylum, NAG specifically stimulated the family *Coriobacteriaceae* (mainly *Collinsella* spp.). NAG also promoted the growth of specific propionate producers (e.g., *Phocaeicola*, *Alistipes*, *Barnesiella*, and *Dialister*), although these effects were milder than those observed with SBI (Figures S2–S4).

The combined treatment SBI_NAG integrated effects of both individual products. SBI_NAG shifted along both PC1 and PC2 in the PCA, reflecting the combination of SBI-associated and NAG-associated compositional changes (Figure 4C and Figures S2–S4). Similar to SBI, the blend promoted *Bacteroidaceae*, *Marinifilaceae*, *Rikenellaceae*, and *Tannerellaceae*, while also maintaining the strong stimulation of *Bifidobacteriaceae*, *Lachnospiraceae*, and *Ruminococcaceae* observed with NAG (Figure 4D–F and Figures S2–S4). Certain taxa (e.g., *Dialisteraceae* and *Barnesiellaceae*) which were already stimulated by both SBI and NAG individually, reached even higher levels in the combined treatment. Overall, SBI_NAG combined beneficial effects of both SBI and NAG into a balanced modulation of a wide range of gut microbiota, aligning with the highest CMS values among all treatments (Figure 4A,B). In contrast, traditional diversity indices failed to detect the broad stimulatory effects of the treatments (Figure S5).

### 3.4. Divergent Metabolic Shifts by SBI and NAG Complemented Each Other When Combined

**Figure 5:**
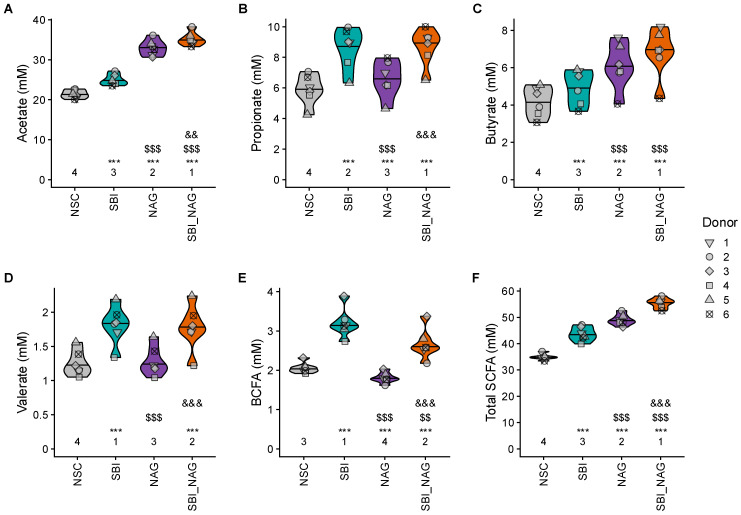
**SBI_NAG combined the individual effects of SBI and NAG on SCFA and BCFA production**. The impact on (**A**) acetate, (**B**) propionate, (**C**) butyrate, (**D**) valerate, (**E**) BCFA, and (**F**) total SCFA production (mM) for the six test subjects. Statistical differences between NSC and individual treatments are indicated with asterisk(s) (*), while differences between SBI and NAG or SBI_NAG are indicated with dollar sign(s) ($) and differences between NAG and SBI_NAG are indicated with ampersand(s) (&). The number of *, $ and & indicates the range of the adjusted *p*-value: one (0.01 < *p*_adjusted_ < 0.05), two (0.001 < *p*_adjusted_ < 0.01), or three symbols (*p*_adjusted_ < 0.001). The ranks of the average values per treatment are shown as well.

The product-specific effects on microbiome composition were mirrored in distinct patterns of metabolite production. All treatments significantly impacted key fermentation parameters, including pH, SCFA (acetate, propionate, butyrate, and valerate), BCFA, and gas production (Figure 5 and Figure S6). However, the specificity of these effects differed between SBI and NAG. SBI primarily enhanced propionate, valerate, and BCFA production, but had no impact on pH. On the other hand, NAG showed a stronger impact on acetate, butyrate, and gas production and significantly reduced BCFA levels. The combined treatment SBI_NAG generally reflected the effects of both its components. Acetate, butyrate, total SCFA, and gas production was significantly higher than SBI and NAG alone. Propionate and valerate levels were similar to the levels observed for SBI, while acidification was similar to the levels observed for NAG.

Additionally, correlation analysis also demonstrated significant positive correlations between TEER (before and after LPS exposure) and SCFA production, including acetate and butyrate, confirming their role in maintaining a healthy gut barrier (Figure S7). Moreover, positive correlations were identified between SCFA production and distinct taxonomic changes. For taxa that increased more strongly with NAG, significant correlations were observed between *Lachnospiraceae*, *Faecalibacterium*, and butyrate levels, as well as between *Lachnospiraceae* and acetate levels (Figure S8A), and between *Bifidobacteriaceae* and butyrate production (Figure S8B). Conversely, propionate-producing Bacteroidota taxa such as *Bacteroidaceae* and *Parabacteroides*, which were specifically augmented by SBI, demonstrated positive correlations with propionate production (Figure S8C).

**Figure 6:**
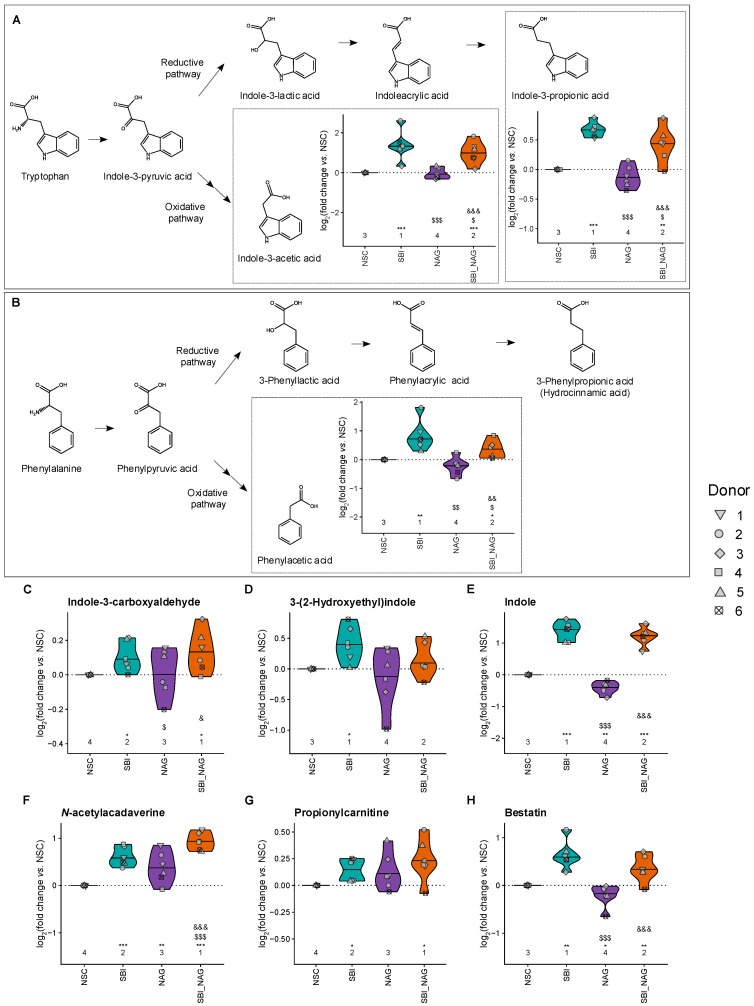
**Stimulation of health-promoting metabolites by SBI and SBI_NAG**. Impact of the test products on the production of (**A**–**E**) metabolites of aromatic amino acids (indoles and phenylacetic acid) and (**F**–**H**) other health-related metabolites, presented as log_2_-transformed fold change relative to NSC for the six test subjects. Statistical differences between NSC and individual treatments are indicated with asterisk(s) (*), while differences between SBI and NAG or SBI_NAG are indicated with dollar sign(s) ($) and differences between NAG and SBI_NAG are indicated with ampersand(s) (&). The number of *, $ and & indicates the range of the adjusted *p*-value: one (0.01 < *p*_adjusted_ < 0.05), two (0.001 < *p*_adjusted_ < 0.01), or three symbols (*p*_adjusted_ < 0.001). The ranks of the average values per treatment are shown as well.

To assess metabolic changes beyond the traditionally studied fermentation parameters, an untargeted metabolomics approach was applied. Fifteen metabolites were significantly affected by at least one treatment (Figure S9). SBI specifically stimulated phenylacetic acid (a phenylalanine derivate) and several indole compounds derived from tryptophan, including indole-3-acetic acid (IAA), indole-3-propionic acid (IPA), indole-3-carboxyaldehyde (I3A), 3-(2-hydroxyethyl)indole (tryptophol), and indole (Figure 6A–E, Figures S9–S10). Significant positive correlations were observed between these indole compounds and various Bacteroidota families which were strongly promoted by SBI (Figure S11). These aromatic amino acid metabolites were unaffected or reduced by NAG, while SBI_NAG enhanced their levels, albeit generally to a lesser extent than SBI alone. A similar trend was observed for bestatin (Figure 6H) and 2-methylbutanoic acid and 2-methylsuccinic acid. In contrast, two lysine-derived metabolites, *N*-acetylcadaverine and propionylcarnitine, were promoted by SBI, NAG, and especially their combination (Figure 6F,G).

## 4. Discussion

This study investigated the impact of SBI, NAG, and their combination on the human gut microbiome and host–microbiome interactions using the ex vivo SIFR^®^ technology. SBI and NAG exerted strong and distinct effects across all parameters investigated as follows: gut barrier integrity, microbial composition, and metabolite production. Notably, their combination SBI_NAG integrated these effects, yielding a superior overall response. This also suggests that the distinct impacts of SBI and NAG likely enable fine-tuning of their ratio within the blend to achieve predictable and desirable outcomes, unlike combinations of prebiotic products with overlapping modes of action. The improvements of gut barrier integrity coincided with the stimulation of beneficial gut microbes and metabolites (extending well beyond traditionally studied SCFA), suggesting a mechanistic link between microbiome modulation and epithelial barrier integrity.

All treatments significantly improved gut barrier integrity, measured as TEER, with the combination SBI_NAG showing the strongest effect under both unstressed and LPS-stressed conditions. These findings are consistent with previous reports on SBI and NAG individually, which have demonstrated their capacity to enhance epithelial integrity [21,27,30,40,41,42]. This study is, however, the first to report on their combined use, revealing complementary effects that suggest compatible modes of action of the protein-based SBI and carbohydrate-based NAG. Given the central role of gut barrier dysfunction in a wide range of diseases, including IBD, metabolic disorders, and auto-immune conditions [4], the observed improvements in barrier function warrant further investigation.

SBI and NAG each induced distinct shifts in microbial composition. Their different biochemical nature (protein *vs*. carbohydrate) translated into unique microbial signatures. SBI primarily enriched propionate-producing taxa within the Bacteroidota phylum, especially the *Bacteroidaceae* family, but also *Rikenellaceae* and *Tannerellaceae*, while additionally stimulating *Anaerotignaceae* and *Dialisteraceae*, both associated with propionate production as well. While its impact on Bacillota_A members was milder, SBI still promoted several taxa from *Lachnospiraceae* and *Butyricicoccaceae*, including several butyrate producers. These findings are consistent with previous ex vivo studies [27,29], which reported similar increases in *Bacteroidaceae* (*Bacteroides* spp., *Phocaeicola vulgatus*), *Tannerellaceae* (*Parabacteroides distasonis*), and *Rikenellaceae* (*Alistipes* spp.), alongside stimulation of *Lachnospiraceae* (e.g., *Dorea* spp.) and *Anaerotignaceae* (*Anaerotignum lactatifermentans*). NAG, on the other hand, strongly promoted butyrate-producing taxa from the families *Ruminococcaceae* and *Lachnospiraceae*, along with other *Lachnospiraceae* members. It also exerted bifidogenic effects, increasing the abundance of *Bifidobacteriaceae*. NAG stimulated certain propionogenic taxa, including *Dialister*, *Phocaeicola*, *Alistipes*, and *Barnesiella*, albeit milder than SBI. While earlier reports on NAG’s impact on microbial composition are limited, a study using a mouse model of ulcerative colitis found a reduction in pathogenic Betaproteobacteria [40], supporting its role in promoting a healthier microbiome. Finally, the combined treatment SBI_NAG integrated effects from both individual products, stimulating a broader range of beneficial taxa. This was reflected in the highest CMS value among all tested conditions for SBI_NAG. Unlike traditional diversity indices, which failed to capture these effects, CMS provided a more accurate measure of microbial responsiveness to prebiotic treatments. Since CMS quantifies the balance between stimulated and suppressed taxa, it is less sensitive to biases introduced by an increase in microbial load, which is often observed upon prebiotic supplementation [54].

The distinct microbial shifts induced by SBI and NAG were mirrored in their fermentation profiles. SBI specifically increased propionate, consistent with its strong stimulation of propionate producers. Acetate, valerate, and butyrate levels also increased, with the latter linked to the mild enrichment of butyrate-producing taxa. As a protein-based compound, SBI additionally elevated BCFA concentrations, typically associated with the fermentation of branched-chain amino acids [57]. Similar fermentation patterns have been reported in previous ex vivo studies with SBI [27,29]. In contrast, this study is the first to report on the impact of NAG supplementation on SCFA production. NAG specifically promoted acetate and butyrate production, consistent with its stimulation of butyrate producers and its bifidogenic effect. Although Bifidobacteria do not produce butyrate directly, they generate acetate and lactate which can subsequently be used for cross-feeding by butyrate producers [58]. The combined treatment SBI_NAG again merged the effects of both individual compounds, resulting in a balanced SCFA profile with elevated levels of acetate, propionate, and butyrate. This highlights the complementary nature of SBI and NAG and may be particularly beneficial given the unique benefits of each individual SCFA [59,60,61,62,63]. Briefly, acetate is metabolized in peripheral tissues after reaching the portal vein. Propionate is taken up by the liver and contributes to metabolic health by promoting satiety, lowering cholesterol, and improving insulin sensitivity. Butyrate is the preferred fuel for epithelial cells and plays a key role in maintaining gut barrier integrity by strengthening tight junctions and stimulating mucin production, as also evidenced in this study by the positive correlations between TEER and butyrate. Valerate, though less studied, has demonstrated antipathogenic activity against *Clostridium difficile* [64] and may inhibit cancer cell growth [65]. BCFA have been associated with beneficial effects, including reduced epithelial permeability [66] and improved insulin sensitivity [67], although their link with proteolytic fermentation also highlights potential risks. Taken together, the balanced SCFA profile with SBI_NAG may contribute to enhanced epithelial function and overall systemic health.

Beyond SCFA production, metabolomics analysis revealed that SBI notably stimulated the production of several health-associated metabolites, particularly those linked to aromatic amino acid catabolism. This included a range of indole derivates from tryptophan (IAA, IPA, I3A, tryptophol, and indole) as well as phenylacetic acid from phenylalanine. These compounds are known for their anti-inflammatory, antioxidative, neuroprotective, and gut barrier-supporting properties [68,69,70,71]. For instance, IPA has been associated with improved cognitive function, reduced risk of type 2 diabetes, and protection against liver and kidney damage [72,73,74], while IAA and I3A contribute to epithelial homeostasis and immune regulation via AhR activation [70,75]. Positive correlations were identified between these indole compounds and Bacteroidota families that are strongly promoted by SBI. Nevertheless, further research is required to elucidate whether these taxa directly contribute to the microbial synthesis of these compounds. SBI also promoted other metabolites such as bestatin (with immunomodulatory and anticancer potential [76,77]) and 2-methylbutanoic acid and 2-methylsuccinic acid (branched-chain fatty acids with possible lipid-lowering and anti-inflammatory effects [78]). Polyamine derivates *N*-acetylcadaverine and propionylcarnitine were stimulated by both SBI and NAG and are associated with cardiovascular health, metabolic regulation, and longevity [79,80,81,82]. As a remark, although these metabolites demonstrated increased production with SBI ex vivo within the colonic environment, their potential systemic health benefits have yet to be further evaluated through in vivo studies.

Finally, while the increase in BCFA and amino acid-derived metabolites upon SBI supplementation may present health benefits, these compounds are also indicative of proteolytic fermentation. Excessive proteolysis is associated with increased production of toxic metabolites like ammonia and hydrogen sulfide, mucosal inflammation, and greater risk of metabolic syndrome, obesity, diabetes and non-alcoholic fatty liver disease [83,84]. The balanced metabolic profile of SBI_NAG, combining proteolytic and saccharolytic fermentation, may mitigate such risks while maximizing health benefits. Further, it is important to note that this study was conducted using an ex vivo fermentation model and cancer-derived cell lines, which, while predictive, do not fully replicate in vivo physiology. Clinical validation is essential to confirm the translational relevance of these findings. Additionally, treatment effects were assessed at a single time point (24 h), as this SIFR^®^ experimental configuration has been shown to generate findings mirroring longer-term changes observed in clinical studies [43]. Follow-up studies should capture the dynamics of taxonomic and metabolic shifts over time to gain mechanistic insights. Moreover, this study simulated full colonic delivery of NAG as no solid clinical data exists on its potential absorption in the upper gastrointestinal tract. Our findings remain relevant if NAG is delivered to the colon via a capsule, even if it is absorbed.

## 5. Conclusions

This study demonstrates that SBI and NAG exert complementary effects on gut health, with their combination yielding the most pronounced improvement in gut barrier integrity. These benefits were accompanied by distinct shifts in microbial composition and metabolite production, suggesting potential mechanistic links. The complementary nature of SBI and NAG supports the development of targeted nutritional strategies aimed at enhancing gut barrier function and microbial metabolic balance. Future work should also focus on optimizing the dose and ratio between SBI and NAG to maximize their benefits in specific health contexts. Further, the preclinical results in this study are exploratory; clinical validation is required to confirm efficacy and safety as well as assess their relevance for individuals with gut barrier impairment or dysbiosis. Additionally, while the current work focuses on functional outcomes, future research should clarify the mechanistic basis of these complementary effects, for example by examining whether SBI- and NAG-derived metabolites synergistically activate signaling networks to strengthen the epithelial barrier.

## Data Availability

The original contributions presented in the study are included in the article/Supplementary Material, further inquiries can be directed to the corresponding author.

## Supplementary Materials

The following supporting information can be downloaded at https://www.mdpi.com/article/10.3390/nu18020210/s1; Figure S1. SBI, NAG and SBI_NAG promoted gut barrier integrity; Figure S2. SBI, NAG and SBI_NAG stimulated specific microbial families; Figure S3. SBI, NAG and SBI_NAG stimulated specific microbial genera; Figure S4. SBI, NAG and SBI_NAG stimulated specific microbial species; Figure S5. Traditional diversity indices do not reflect the stimulatory effects of SBI, NAG and SBI_NAG on a broad range of microbiota; Figure S6. SBI_NAG combined the individual effects of SBI and NAG on pH and gas production; Figure S7. Gut barrier integrity (TEER) positively correlated with SCFA production; Figure S8. SCFA production correlated with specific taxa stimulated by SBI and/or NAG; Figure S9. SBI, NAG and SBI_NAG affected specific metabolites; Figure S10. Representative MS/MS spectrum of the tryptophan derivative indole-3-propionic acid (IPA), indole-3-acetic acid (IAA) and indole-3-carboxyaldehyde (I3A); Figure S11. Bacteroidota families positively correlated with the production of various indoles.

## Abbreviations

The following abbreviations are used in this manuscript:

BCFA	Branched-chain fatty acids
CMS	Community modulation score
FDR	False discovery rate
I3A	Indole-3-carboxyaldehyde
IAA	Indole-3-acetic acid
IBD	Inflammatory bowel disease
IPA	Indole-3-propionic acid
LPS	Lipopolysaccharide
NAG	*N*-acetylglucosamine
NSC	No-substrate control
PCA	Principal component analysis
SBI	Serum-derived bovine immunoglobulin
SCFA	Short-chain fatty acids
SIFR^®^	Systemic intestinal fermentation research
TEER	Transepithelial electrical resistance

## References

[1] Hou K., Wu Z.-X., Chen X.-Y., Wang J.-Q., Zhang D., Xiao C., Zhu D., Koya J.B., Wei L., Li J. (2022). Microbiota in Health and Diseases. Signal Transduct. Target. Ther..

[2] Khalil M., Di Ciaula A., Mahdi L., Jaber N., Di Palo D.M., Graziani A., Baffy G., Portincasa P. (2024). Unraveling the Role of the Human Gut Microbiome in Health and Diseases. Microorganisms.

[3] Martel J., Chang S.-H., Ko Y.-F., Hwang T.-L., Young J.D., Ojcius D.M. (2022). Gut Barrier Disruption and Chronic Disease. Trends Endocrinol. Metab..

[4] Santilli A., Stefanopoulos S., Cresci G.A.M. (2022). The Gut Barrier and Chronic Diseases. Curr. Opin. Clin. Nutr. Metab. Care.

[5] Macura B., Kiecka A., Szczepanik M. (2024). Intestinal Permeability Disturbances: Causes, Diseases and Therapy. Clin. Exp. Med..

[6] Michielan A., D’Incà R. (2015). Intestinal Permeability in Inflammatory Bowel Disease: Pathogenesis, Clinical Evaluation, and Therapy of Leaky Gut. Mediat. Inflamm..

[7] Valitutti F., Fasano A. (2019). Breaking Down Barriers: How Understanding Celiac Disease Pathogenesis Informed the Development of Novel Treatments. Dig. Dis. Sci..

[8] Gecse K., Róka R., Séra T., Rosztóczy A., Annaházi A., Izbéki F., Nagy F., Molnár T., Szepes Z., Pávics L. (2011). Leaky Gut in Patients with Diarrhea-Predominant Irritable Bowel Syndrome and Inactive Ulcerative Colitis. Digestion.

[9] Lau W.L., Tran T., Rhee C.M., Kalantar-Zadeh K., Vaziri N.D. (2021). Diabetes and the Gut Microbiome. Semin. Nephrol..

[10] Portincasa P., Bonfrate L., Khalil M., Angelis M.D., Calabrese F.M., D’Amato M., Wang D.Q.-H., Di Ciaula A. (2021). Intestinal Barrier and Permeability in Health, Obesity and NAFLD. Biomedicines.

[11] Ma L., Morel L. (2022). Loss of Gut Barrier Integrity in Lupus. Front. Immunol..

[12] Lobiuc A., Groppa L., Chislari L., Russu E., Homitchi M., Ciorescu C., Hamamah S., Bran I.C., Covasa M. (2025). Gut Microbiota and Ankylosing Spondylitis: Current Insights and Future Challenges. Microb. Cell.

[13] Polak K., Bergler-Czop B., Szczepanek M., Wojciechowska K., Frątczak A., Kiss N. (2021). Psoriasis and Gut Microbiome—Current State of Art. Int. J. Mol. Sci..

[14] Niewiem M., Grzybowska-Chlebowczyk U. (2022). Intestinal Barrier Permeability in Allergic Diseases. Nutrients.

[15] Hijazi Z., Molla A.M., Al-Habashi H., Muawad W.M.R.A., Molla A.M., Sharma P.N. (2004). Intestinal Permeability Is Increased in Bronchial Asthma. Arch. Dis. Child..

[16] Blicharz L., Samborowska E., Zagożdżon R., Bukowska-Ośko I., Czuwara J., Zych M., Roszczyk A., Perlejewski K., Makowska K., Nowaczyk J. (2025). Severity of Atopic Dermatitis Is Associated with Gut-Derived Metabolites and Leaky Gut-Related Biomarkers. Sci. Rep..

[17] Fattorusso A., Di Genova L., Dell’Isola G.B., Mencaroni E., Esposito S. (2019). Autism Spectrum Disorders and the Gut Microbiota. Nutrients.

[18] Lee S.-Y., Li S.-C., Yang C.-Y., Kuo H.-C., Chou W.-J., Wang L.-J. (2023). Gut Leakage Markers and Cognitive Functions in Patients with Attention-Deficit/Hyperactivity Disorder. Children.

[19] Liu L., Wang H., Chen X., Zhang Y., Zhang H., Xie P. (2023). Gut Microbiota and Its Metabolites in Depression: From Pathogenesis to Treatment. eBioMedicine.

[20] Stadlbauer V., Engertsberger L., Komarova I., Feldbacher N., Leber B., Pichler G., Fink N., Scarpatetti M., Schippinger W., Schmidt R. (2020). Dysbiosis, Gut Barrier Dysfunction and Inflammation in Dementia: A Pilot Study. BMC Geriatr..

[21] Petschow B.W., Burnett B.P., Shaw A.L., Weaver E.M., Klein G.L. (2015). Dietary Requirement for Serum-Derived Bovine Immunoglobulins in the Clinical Management of Patients with Enteropathy. Dig. Dis. Sci..

[22] Henderson A.L., Brand M.W., Darling R.J., Maas K.J., Detzel C.J., Hostetter J., Wannemuehler M.J., Weaver E.M. (2015). Attenuation of Colitis by Serum-Derived Bovine Immunoglobulin/Protein Isolate in a Defined Microbiota Mouse Model. Dig. Dis. Sci..

[23] Detzel C.J., Horgan A., Henderson A.L., Petschow B.W., Warner C.D., Maas K.J., Weaver E.M. (2015). Bovine Immunoglobulin/Protein Isolate Binds Pro-Inflammatory Bacterial Compounds and Prevents Immune Activation in an Intestinal Co-Culture Model. PLoS ONE.

[24] Petschow B.W., Burnett B., Shaw A.L., Weaver E.M., Klein G.L. (2014). Serum-Derived Bovine Immunoglobulin/Protein Isolate: Postulated Mechanism of Action for Management of Enteropathy. Clin. Exp. Gastroenterol..

[25] Petschow B.W., Blikslager A.T., Weaver E.M., Campbell J.M., Polo J., Shaw A.L., Burnett B.P., Klein G.L., Rhoads J.M. (2014). Bovine Immunoglobulin Protein Isolates for the Nutritional Management of Enteropathy. World J. Gastroenterol..

[26] Ulfman L.H., Leusen J.H.W., Savelkoul H.F.J., Warner J.O., van Neerven R.J.J. (2018). Effects of Bovine Immunoglobulins on Immune Function, Allergy, and Infection. Front. Nutr..

[27] Van den Abbeele P., Kunkler C.N., Poppe J., Rose A., van Hengel I.A.J., Baudot A., Warner C.D. (2024). Serum-Derived Bovine Immunoglobulin Promotes Barrier Integrity and Lowers Inflammation for 24 Human Adults Ex Vivo. Nutrients.

[28] Hazan S., Bao G., Vidal A., Sfera A. (2024). Gut Microbiome Alterations Following Oral Serum-Derived Bovine Immunoglobulin Administration in the Management of Dysbiosis. Cureus.

[29] Van den Abbeele P., Detzel C., Rose A., Deyaert S., Baudot A., Warner C. (2023). Serum-Derived Bovine Immunoglobulin Stimulates SCFA Production by Specific Microbes in the Ex Vivo SIFR^®^ Technology. Microorganisms.

[30] Utay N.S., Somasunderam A., Hinkle J.E., Petschow B.W., Detzel C.J., Somsouk M., Fichtenbaum C.J., Weaver E.M., Shaw A.L., Asmuth D.M. (2019). Serum Bovine Immunoglobulins Improve Inflammation and Gut Barrier Function in Persons with HIV and Enteropathy on Suppressive ART. Pathog. Immun..

[31] Liaquat H., Ashat M., Stocker A., McElmurray L., Beatty K., Abell T.L., Dryden G. (2018). Clinical Efficacy of Serum-Derived Bovine Immunoglobulin in Patients with Refractory Inflammatory Bowel Disease. Am. J. Med. Sci..

[32] Shafran I., Burgunder P., Wei D., Young H.E., Klein G., Burnett B.P. (2015). Management of Inflammatory Bowel Disease with Oral Serum-Derived Bovine Immunoglobulin. Ther. Adv. Gastroenterol..

[33] Valentin N., Camilleri M., Carlson P., Harrington S.C., Eckert D., O’Neill J., Burton D., Chen J., Shaw A.L., Acosta A. (2017). Potential Mechanisms of Effects of Serum-Derived Bovine Immunoglobulin/Protein Isolate Therapy in Patients with Diarrhea-Predominant Irritable Bowel Syndrome. Physiol. Rep..

[34] Wilson D., Evans M., Weaver E., Shaw A.L., Klein G.L. (2013). Evaluation of Serum-Derived Bovine Immunoglobulin Protein Isolate in Subjects with Diarrhea-Predominant Irritable Bowel Syndrome. Clin. Med. Insights Gastroenterol..

[35] Shaw A.L., Tomanelli A., Bradshaw T.P., Petschow B.W., Burnett B.P. (2017). Impact of Serum-Derived Bovine Immunoglobulin/Protein Isolate Therapy on Irritable Bowel Syndrome and Inflammatory Bowel Disease: A Survey of Patient Perspective. Patient Prefer. Adherence.

[36] Asmuth D.M., Ma Z.-M., Albanese A., Sandler N.G., Devaraj S., Knight T.H., Flynn N.M., Yotter T., Garcia J.-C., Tsuchida E. (2013). Oral Serum-Derived Bovine Immunoglobulin Improves Duodenal Immune Reconstitution and Absorption Function in Patients with HIV Enteropathy. AIDS.

[37] Utay N.S., Güerri-Fernández R., Gharakhanian S., Asmuth D.M., Contreras M., Kunkler C., Detzel C.J., Warner C.D. (2024). Serum-Derived Bovine Immunoglobulin Treatment in COVID-19 Is Associated with Faster Resolution of Symptoms: A Randomized Pilot Clinical Trial. J. Med. Virol..

[38] Rawat P.S., Seyed Hameed A.S., Meng X., Liu W. (2022). Utilization of Glycosaminoglycans by the Human Gut Microbiota: Participating Bacteria and Their Enzymatic Machineries. Gut Microbes.

[39] Fekete E., Buret A.G. (2023). The Role of Mucin O-Glycans in Microbiota Dysbiosis, Intestinal Homeostasis, and Host-Pathogen Interactions. Am. J. Physiol.-Gastrointest. Liver Physiol..

[40] Choi S.-I., Shin Y.C., Lee J.S., Yoon Y.C., Kim J.M., Sung M.-K. (2023). *N*-Acetylglucosamine and Its Dimer Ameliorate Inflammation in Murine Colitis by Strengthening the Gut Barrier Function. Food Funct..

[41] Liu Y., Xu W., Liu L., Guo L., Deng Y., Liu J. (2012). N-Acetyl Glucosamine Improves Intestinal Mucosal Barrier Function in Rat. Bangladesh J. Pharmacol..

[42] Salvatore S., Heuschkel R., Tomlin S., Davies S.E., Edwards S., Walker-Smith J.A., French I., Murch S.H. (2000). A Pilot Study of N-Acetyl Glucosamine, a Nutritional Substrate for Glycosaminoglycan Synthesis, in Paediatric Chronic Inflammatory Bowel Disease. Aliment. Pharmacol. Ther..

[43] Van den Abbeele P., Deyaert S., Thabuis C., Perreau C., Bajic D., Wintergerst E., Joossens M., Firrman J., Walsh D., Baudot A. (2023). Bridging Preclinical and Clinical Gut Microbiota Research Using the Ex Vivo SIFR^®^ Technology. Front. Microbiol..

[44] Brodkorb A., Egger L., Alminger M., Alvito P., Assunção R., Ballance S., Bohn T., Bourlieu-Lacanal C., Boutrou R., Carrière F. (2019). INFOGEST Static In Vitro Simulation of Gastrointestinal Food Digestion. Nat. Protoc..

[45] Van den Abbeele P., Deyaert S., Albers R., Baudot A., Mercenier A. (2023). Carrot RG-I Reduces Interindividual Differences between 24 Adults through Consistent Effects on Gut Microbiota Composition and Function Ex Vivo. Nutrients.

[46] Doneanu C.E., Chen W., Mazzeo J.R. (2011). UPLC/MS Monitoring of Water-Soluble Vitamin Bs in Cell Culture Media in Minutes. Water Application Note. https://www.waters.com/nextgen/us/en/library/application-notes/2011/uplc-ms-monitoring-water-soluble-vitamin-bs-cell-culture-media-minutes.html?srsltid=AfmBOooiPZsveLaDnOYiW38sfg1PpTZbEzgh_RBWQnV0UX1IBr4lgzEf.

[47] Adams K.J., Pratt B., Bose N., Dubois L.G., John-Williams L., Perrott K.M., Ky K., Kapahi P., Sharma V., MacCoss M.J. (2020). Skyline for Small Molecules: A Unifying Software Package for Quantitative Metabolomics. J. Proteome Res..

[48] Agarwal K., Maki K.A., Vizioli C., Carnell S., Goodman E., Hurley M., Harris C., Colwell R., Steele K., Joseph P.V. (2022). The Neuro-Endo-Microbio-Ome Study: A Pilot Study of Neurobiological Alterations Pre- Versus Post-Bariatric Surgery. Biol. Res. Nurs..

[49] Hasan N.A., Young B.A., Minard-Smith A.T., Saeed K., Li H., Heizer E.M., McMillan N.J., Isom R., Abdullah A.S., Bornman D.M. (2014). Microbial Community Profiling of Human Saliva Using Shotgun Metagenomic Sequencing. PLoS ONE.

[50] Srinivasan B., Kolli A.R., Esch M.B., Abaci H.E., Shuler M.L., Hickman J.J. (2015). TEER Measurement Techniques for In Vitro Barrier Model Systems. J. Lab. Autom..

[51] Husson F., Josse J., Le S., Mazet J. (2022). FactoMineR: Multivariate Exploratory Data Analysis and Data Mining. https://cran.r-project.org/web/packages/FactoMineR/FactoMineR.pdf.

[52] Brooks M.E., Kristensen K., van Benthem K.J., Magnusson A., Berg C.W., Nielsen A., Skaug H.J., Mächler M., Bolker B.M. (2017). glmmTMB Balances Speed and Flexibility Among Packages for Zero-Inflated Generalized Linear Mixed Modeling. R J..

[53] Benjamini Y., Hochberg Y. (1995). Controlling the False Discovery Rate: A Practical and Powerful Approach to Multiple Testing. J. R. Stat. Soc. Ser. B (Methodol.).

[54] Tintoré M., Cuñé J., Vu L.D., Poppe J., Van den Abbeele P., Baudot A., de Lecea C. (2024). A Long-Chain Dextran Produced by *Weissella cibaria* Boosts the Diversity of Health-Related Gut Microbes Ex Vivo. Biology.

[55] Arumugam M., Raes J., Pelletier E., Le Paslier D., Yamada T., Mende D.R., Fernandes G.R., Tap J., Bruls T., Batto J.-M. (2011). Enterotypes of the Human Gut Microbiome. Nature.

[56] Wu G.D., Chen J., Hoffmann C., Bittinger K., Chen Y.-Y., Keilbaugh S.A., Bewtra M., Knights D., Walters W.A., Knight R. (2011). Linking Long-Term Dietary Patterns with Gut Microbial Enterotypes. Science.

[57] McCann J.R., Rawls J.F. (2023). Essential Amino Acid Metabolites as Chemical Mediators of Host-Microbe Interaction in the Gut. Annu. Rev. Microbiol..

[58] Rivière A., Selak M., Lantin D., Leroy F., De Vuyst L. (2016). Bifidobacteria and Butyrate-Producing Colon Bacteria: Importance and Strategies for Their Stimulation in the Human Gut. Front. Microbiol..

[59] Blaak E.E., Canfora E.E., Theis S., Frost G., Groen A.K., Mithieux G., Nauta A., Scott K., Stahl B., van Harsselaar J. (2020). Short Chain Fatty Acids in Human Gut and Metabolic Health. Benef. Microbes.

[60] Xiong R.-G., Zhou D.-D., Wu S.-X., Huang S.-Y., Saimaiti A., Yang Z.-J., Shang A., Zhao C.-N., Gan R.-Y., Li H.-B. (2022). Health Benefits and Side Effects of Short-Chain Fatty Acids. Foods.

[61] Mansuy-Aubert V., Ravussin Y. (2023). Short Chain Fatty Acids: The Messengers from down Below. Front. Neurosci..

[62] Facchin S., Bertin L., Bonazzi E., Lorenzon G., De Barba C., Barberio B., Zingone F., Maniero D., Scarpa M., Ruffolo C. (2024). Short-Chain Fatty Acids and Human Health: From Metabolic Pathways to Current Therapeutic Implications. Life.

[63] Silva Y.P., Bernardi A., Frozza R.L. (2020). The Role of Short-Chain Fatty Acids From Gut Microbiota in Gut-Brain Communication. Front. Endocrinol..

[64] McDonald J.A.K., Mullish B.H., Pechlivanis A., Liu Z., Brignardello J., Kao D., Holmes E., Li J.V., Clarke T.B., Thursz M.R. (2018). Inhibiting Growth of Clostridioides Difficile by Restoring Valerate, Produced by the Intestinal Microbiota. Gastroenterology.

[65] Hinnebusch B.F., Meng S., Wu J.T., Archer S.Y., Hodin R.A. (2002). The Effects of Short-Chain Fatty Acids on Human Colon Cancer Cell Phenotype Are Associated with Histone Hyperacetylation. J. Nutr..

[66] Blachier F., Mariotti F., Huneau J.F., Tomé D. (2007). Effects of Amino Acid-Derived Luminal Metabolites on the Colonic Epithelium and Physiopathological Consequences. Amino Acids.

[67] Heimann E., Nyman M., Pålbrink A.-K., Lindkvist-Petersson K., Degerman E. (2016). Branched Short-Chain Fatty Acids Modulate Glucose and Lipid Metabolism in Primary Adipocytes. Adipocyte.

[68] Negatu D.A., Gengenbacher M., Dartois V., Dick T. (2020). Indole Propionic Acid, an Unusual Antibiotic Produced by the Gut Microbiota, with Anti-Inflammatory and Antioxidant Properties. Front. Microbiol..

[69] Chyan Y.-J., Poeggeler B., Omar R.A., Chain D.G., Frangione B., Ghiso J., Pappolla M.A. (1999). Potent Neuroprotective Properties against the Alzheimer β-Amyloid by an Endogenous Melatonin-Related Indole Structure, Indole-3-Propionic Acid*. J. Biol. Chem..

[70] Hendrikx T., Schnabl B. (2019). Indoles: Metabolites Produced by Intestinal Bacteria Capable of Controlling Liver Disease Manifestation. J. Intern. Med..

[71] Li X., Zhang B., Hu Y., Zhao Y. (2021). New Insights Into Gut-Bacteria-Derived Indole and Its Derivatives in Intestinal and Liver Diseases. Front. Pharmacol..

[72] Kim C.-S., Jung S., Hwang G.-S., Shin D.-M. (2023). Gut Microbiota Indole-3-Propionic Acid Mediates Neuroprotective Effect of Probiotic Consumption in Healthy Elderly: A Randomized, Double-Blind, Placebo-Controlled, Multicenter Trial and In Vitro Study. Clin. Nutr..

[73] de Mello V.D., Paananen J., Lindström J., Lankinen M.A., Shi L., Kuusisto J., Pihlajamäki J., Auriola S., Lehtonen M., Rolandsson O. (2017). Indolepropionic Acid and Novel Lipid Metabolites Are Associated with a Lower Risk of Type 2 Diabetes in the Finnish Diabetes Prevention Study. Sci. Rep..

[74] Jiang H., Chen C., Gao J. (2023). Extensive Summary of the Important Roles of Indole Propionic Acid, a Gut Microbial Metabolite in Host Health and Disease. Nutrients.

[75] Li M., Ding Y., Wei J., Dong Y., Wang J., Dai X., Yan J., Chu F., Zhang K., Meng F. (2024). Gut Microbiota Metabolite Indole-3-Acetic Acid Maintains Intestinal Epithelial Homeostasis through Mucin Sulfation. Gut Microbes.

[76] Kim H.W., Ko M.-K., Park S.H., Shin S., Kim S.-M., Park J.-H., Lee M.J. (2023). Bestatin, A Pluripotent Immunomodulatory Small Molecule, Drives Robust and Long-Lasting Immune Responses as an Adjuvant in Viral Vaccines. Vaccines.

[77] Ma Y., Yang X., Pan P., Yang J., Wu X., Wang D., Gao H. (2024). Bestatin Attenuates Breast Cancer Stemness by Targeting Puromycin-Sensitive Aminopeptidase. Discov. Oncol..

[78] Gozdzik P., Magkos F., Sledzinski T., Mika A. (2023). Monomethyl Branched-Chain Fatty Acids: Health Effects and Biological Mechanisms. Prog. Lipid Res..

[79] Muñoz-Esparza N.C., Latorre-Moratalla M.L., Comas-Basté O., Toro-Funes N., Veciana-Nogués M.T., Vidal-Carou M.C. (2019). Polyamines in Food. Front. Nutr..

[80] Soda K., Dobashi Y., Kano Y., Tsujinaka S., Konishi F. (2009). Polyamine-Rich Food Decreases Age-Associated Pathology and Mortality in Aged Mice. Exp. Gerontol..

[81] Soda K., Kano Y., Chiba F., Koizumi K., Miyaki Y. (2013). Increased Polyamine Intake Inhibits Age-Associated Alteration in Global DNA Methylation and 1,2-Dimethylhydrazine-Induced Tumorigenesis. PLoS ONE.

[82] Mingorance C., Duluc L., Chalopin M., Simard G., Ducluzeau P.-H., Herrera M.D., de Sotomayor M.A., Andriantsitohaina R. (2012). Propionyl-L-Carnitine Corrects Metabolic and Cardiovascular Alterations in Diet-Induced Obese Mice and Improves Liver Respiratory Chain Activity. PLoS ONE.

[83] Diether N.E., Willing B.P. (2019). Microbial Fermentation of Dietary Protein: An Important Factor in Diet–Microbe–Host Interaction. Microorganisms.

[84] Peled S., Livney Y.D. (2021). The Role of Dietary Proteins and Carbohydrates in Gut Microbiome Composition and Activity: A Review. Food Hydrocoll..

