# Supplementary material for: N-Acetylglucosamine and Immunoglobulin Strengthen Gut Barrier Integrity via Complementary Microbiome Modulation
Source: Nutrients. 2026 Jan 9;18(2):210. doi: 10.3390/nu18020210 (PMC12845043; doi:10.3390/nu18020210)
Supplement: Supplementary file 1 [file nutrients-18-00210-s001.zip › nutrients-4030478-supplementary.pdf]

nutrients

Supplementary Materials

N-Acetylglucosamine and Immunoglobulin Strengthen Gut Barrier Integrity Via Complementary Microbiome Modulation

Emma De Beul[1], Jasmine Heyse [1], Michael Jurgelewicz [2,3], Aurélien Baudot [1], Lam Dai Vu [1] and Pieter Van den Abbeele [1,*]

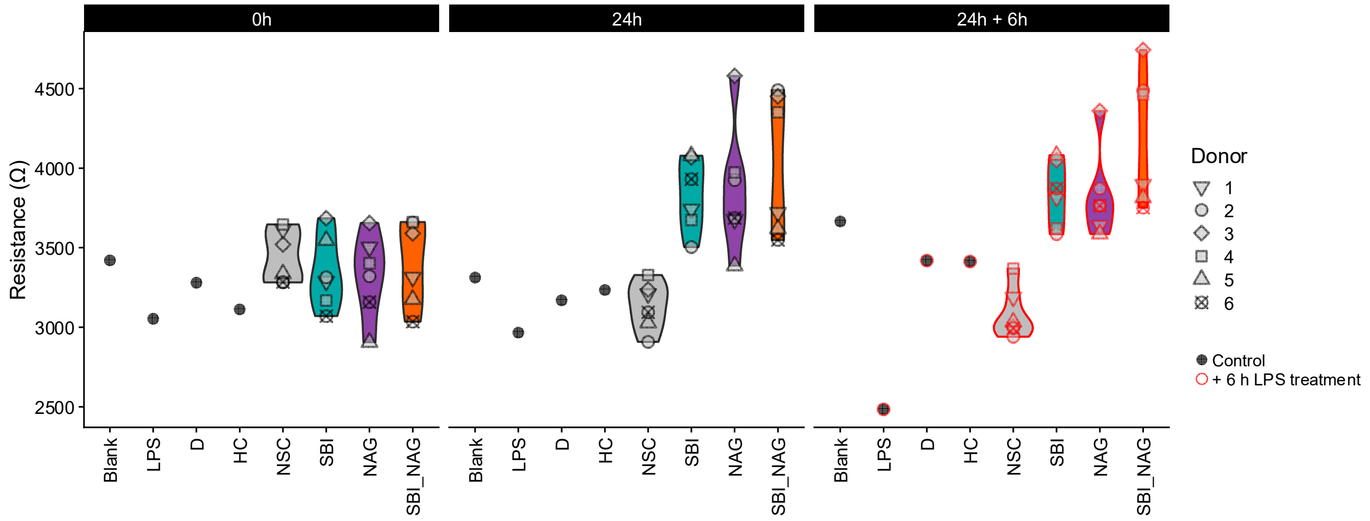

Figure S1. SBI, NAG and SBI_NAG promoted gut barrier integrity. Transepithelial electrical resistance (TEER) (Ω) was used as a measure of gut barrier integrity and was measured at baseline (0h), under unstressed condition after 24h incubation with SIFR®-derived colonic samples, and under stressed conditions, following an additional 6h treatment with lipopolysaccharide (LPS). Treatments samples are based on SIFR®-derived colonic samples from six test subjects. Four control samples (*n* = 1) were also included in the visualization.

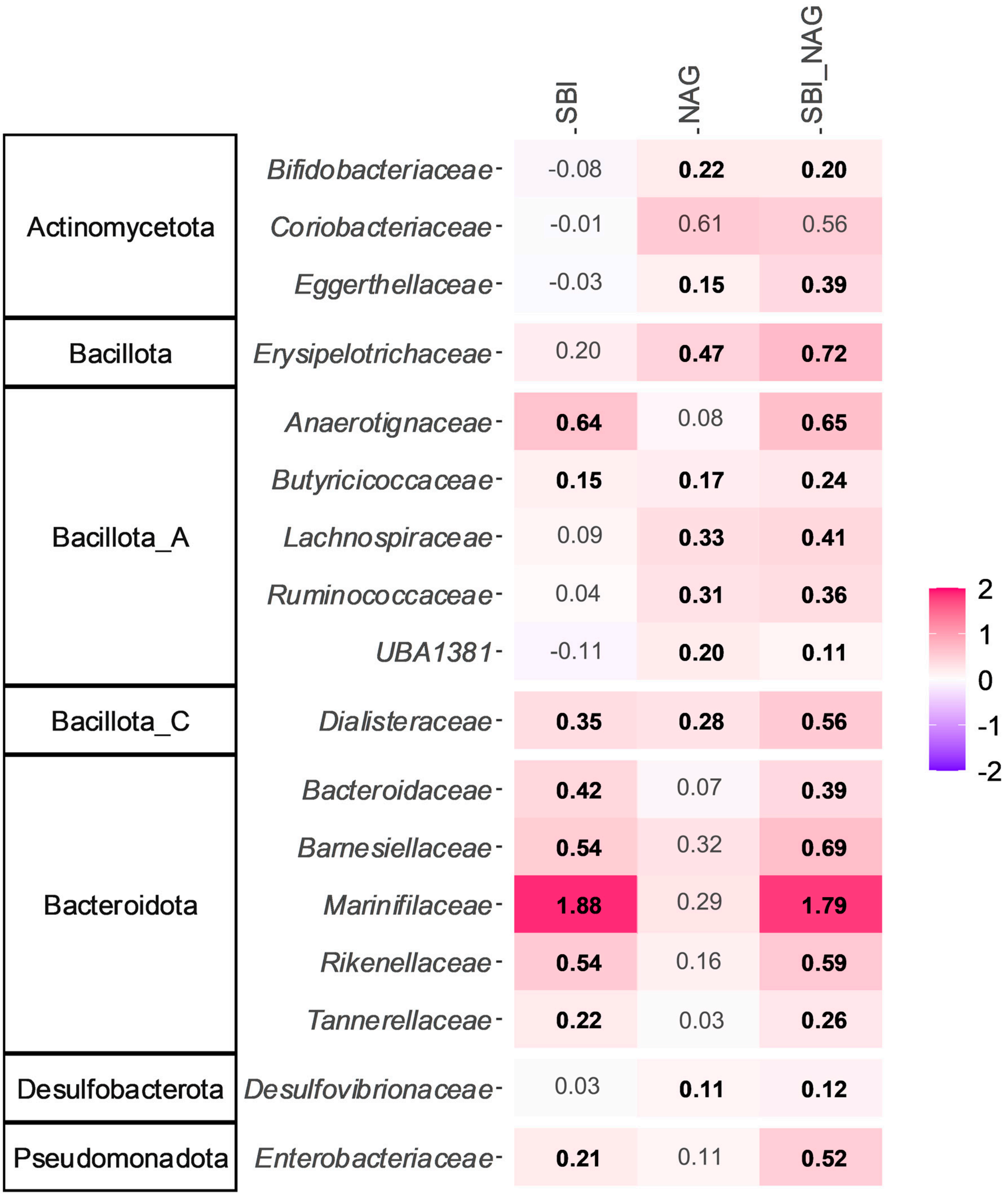

Figure S2. SBI, NAG and SBI_NAG stimulated specific microbial families. The heatmap displays significantly affected families, expressed as log_2 fold change in cell density (cells/mL) compared to the untreated control (NSC) at 24h of incubation in the SIFR® technology (*n* = 6). Bold numbers indicate significant treatment effects. The corresponding phyla are highlighted on the left.

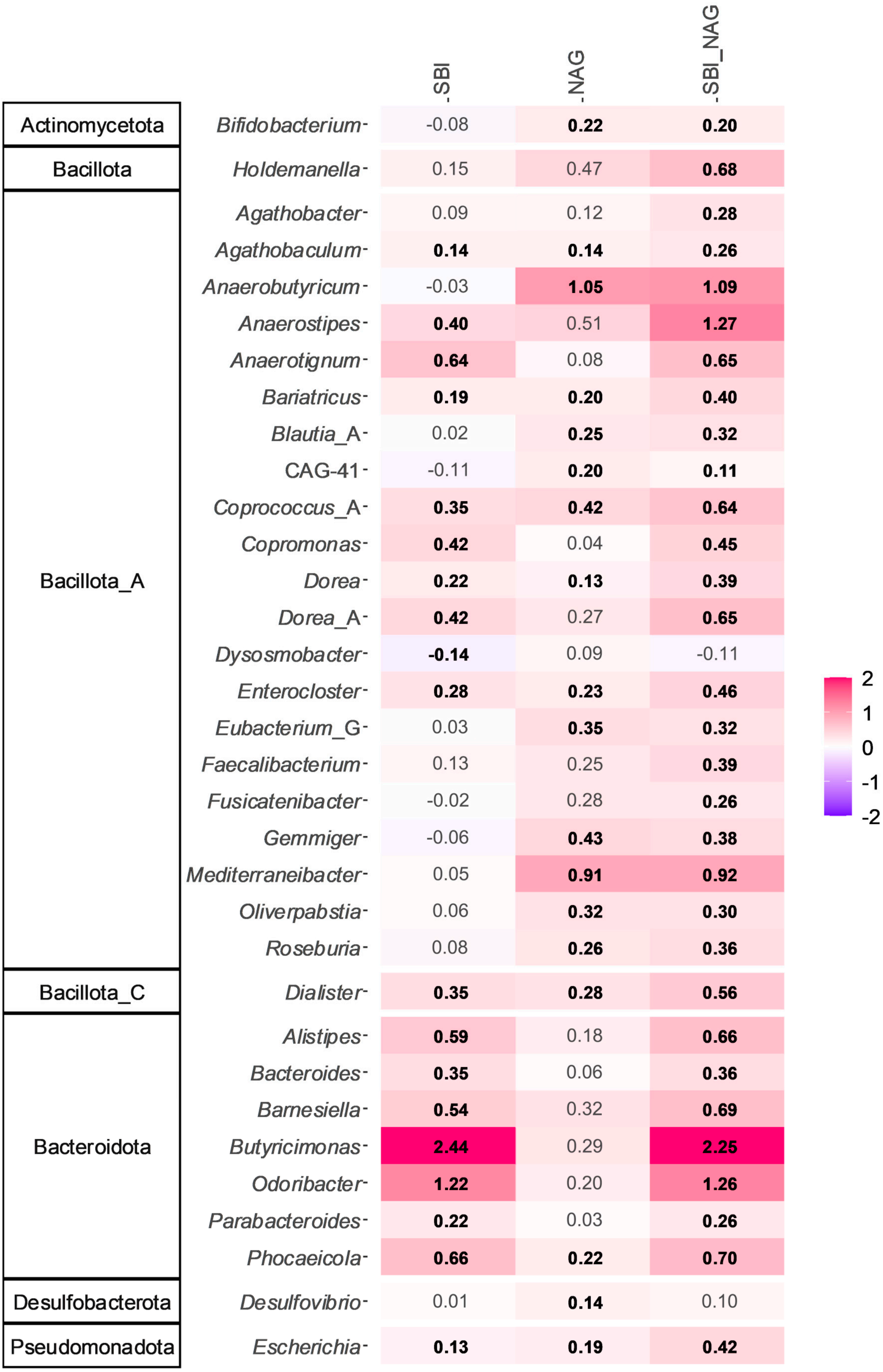

Figure S3. SBI, NAG and SBI_NAG stimulated specific microbial genera. The heatmap displays significantly affected genera, expressed as $\log_2$ fold change in cell density (cells/mL) compared to the untreated control (NSC) at 24h of incubation in the SIFR® technology (n = 6). Bold numbers indicate significant treatment effects. The corresponding phyla are highlighted on the left.

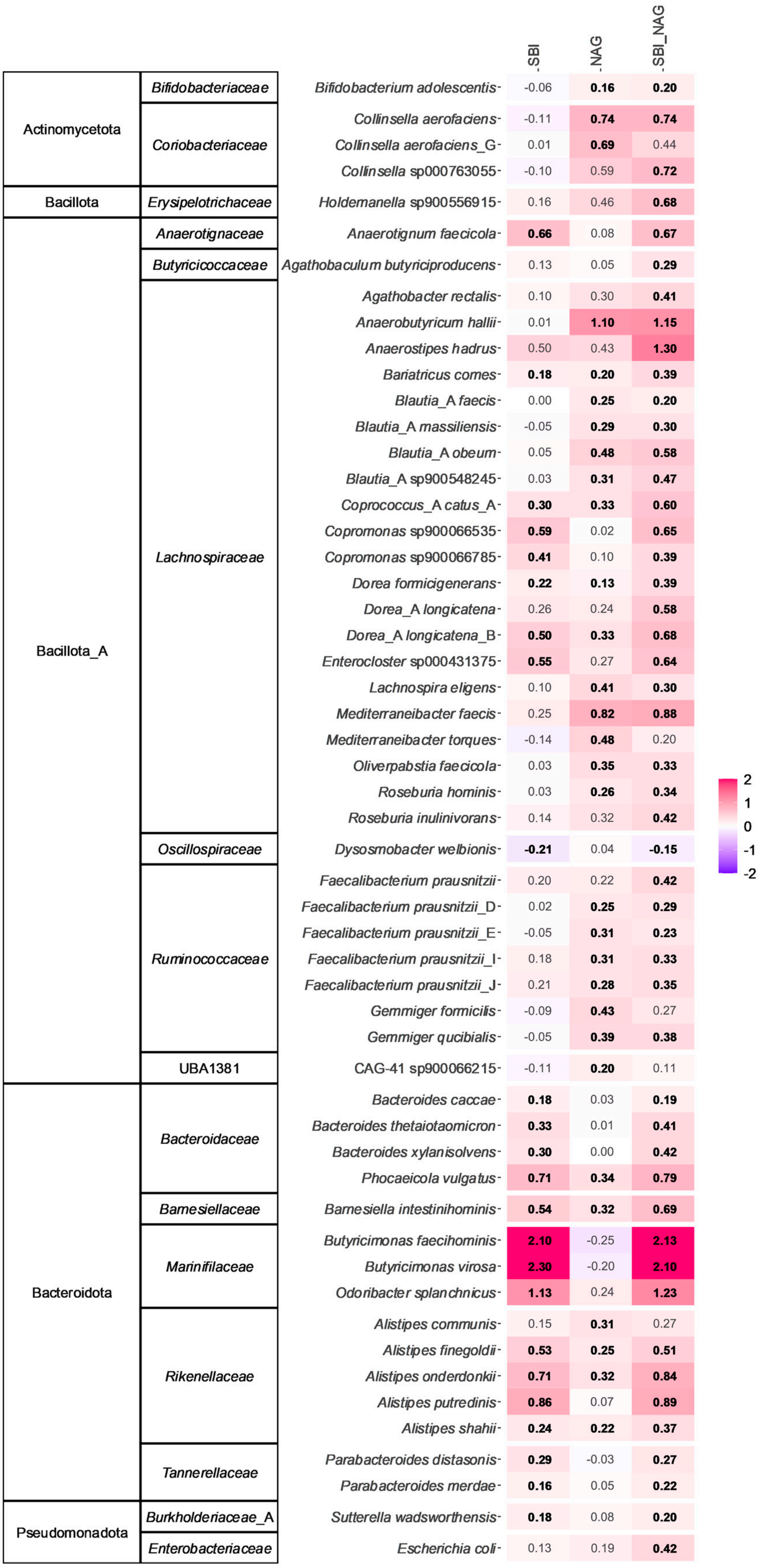

Figure S4. SBI, NAG and SBI_NAG stimulated specific microbial species. The heatmap displays significantly affected species, expressed as log_2 fold change in cell density (cells/mL) compared to the untreated control (NSC) at 24h of incubation in the SIFR® technology (n = 6). Bold numbers indicate significant treatment effects. The corresponding phyla and families are highlighted on the left.

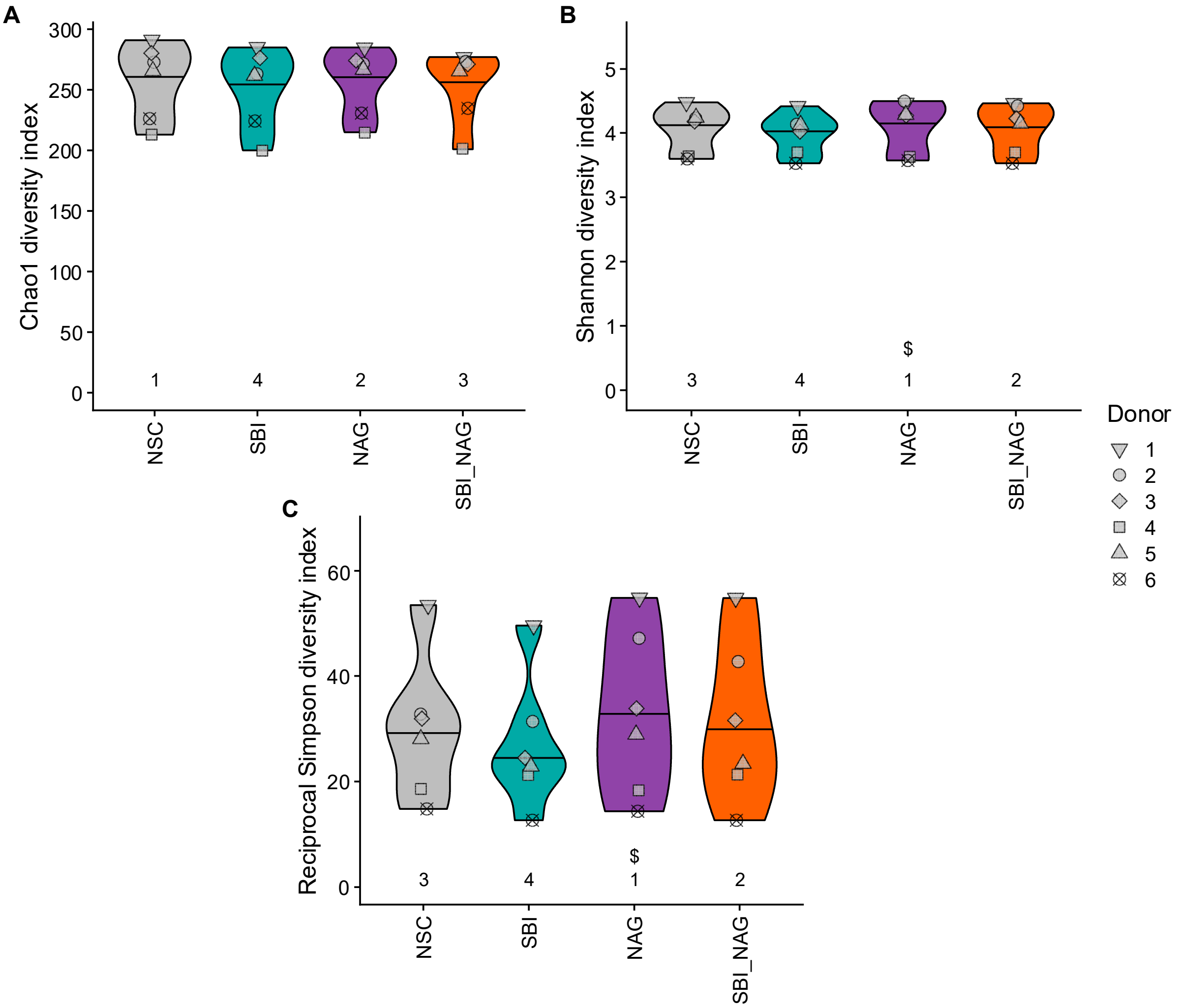

Figure S5. Traditional diversity indices do not reflect the stimulatory effects of SBI, NAG and SBI_NAG on a broad range of microbiota. Impact of SBI, NAG and SBI_NAG on (**A**) Chao1, (**B**) Shannon and (**C**) reciprocal Simpson diversity indices (n = 6). Statistical differences between the untreated control (NSC) and individual treatments are indicated with * ($0.01 < p_{adjusted} < 0.05$), ** ($0.001 < p_{adjusted} < 0.01$) or *** ($p_{adjusted} < 0.001$), while differences between SBI and NAG or SBI_NAG are indicated with $/$$/$$ and differences between NAG and SBI_NAG are indicated with &/&&/&&&. The ranks of the average values per treatment are shown as well.

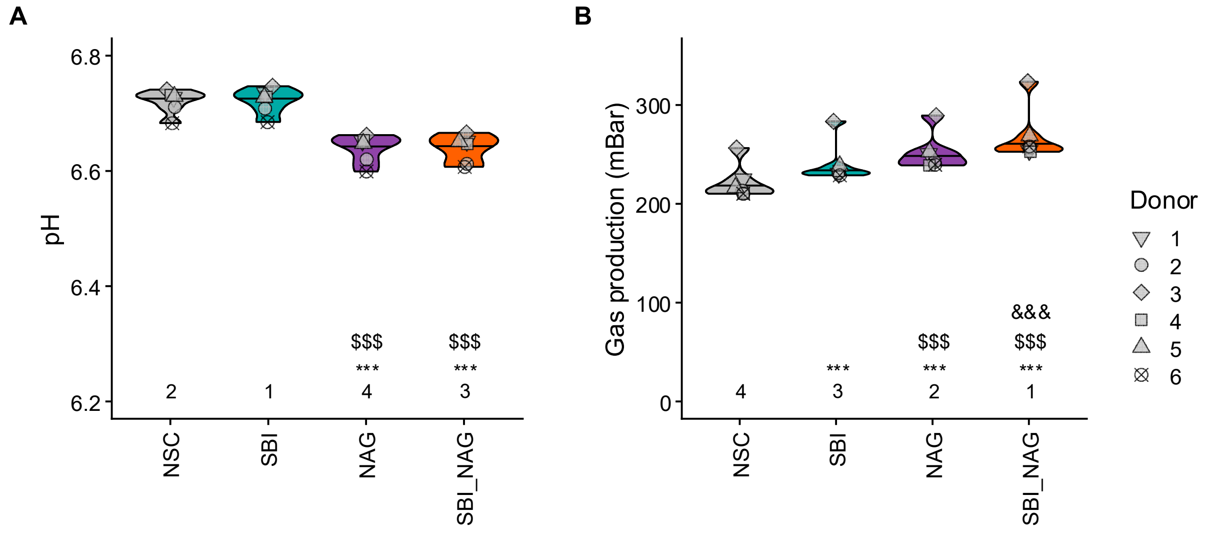

Figure S6. SBI_NAG combined the individual effects of SBI and NAG on pH and gas production. The impact on (**A**) pH and (**B**) gas production (mBar) (n = 6). Statistical differences between NSC and individual treatments are indicated with * ($0.01 < p_{adjusted} < 0.05$), ** ($0.001 < p_{adjusted} < 0.01$) or *** ($p_{adjusted} < 0.001$), while differences between SBI and NAG or SBI_NAG are indicated with $/$$/$$ and differences between NAG and SBI_NAG are indicated with &/&&/&&&. The ranks of the average values per treatment are shown as well.

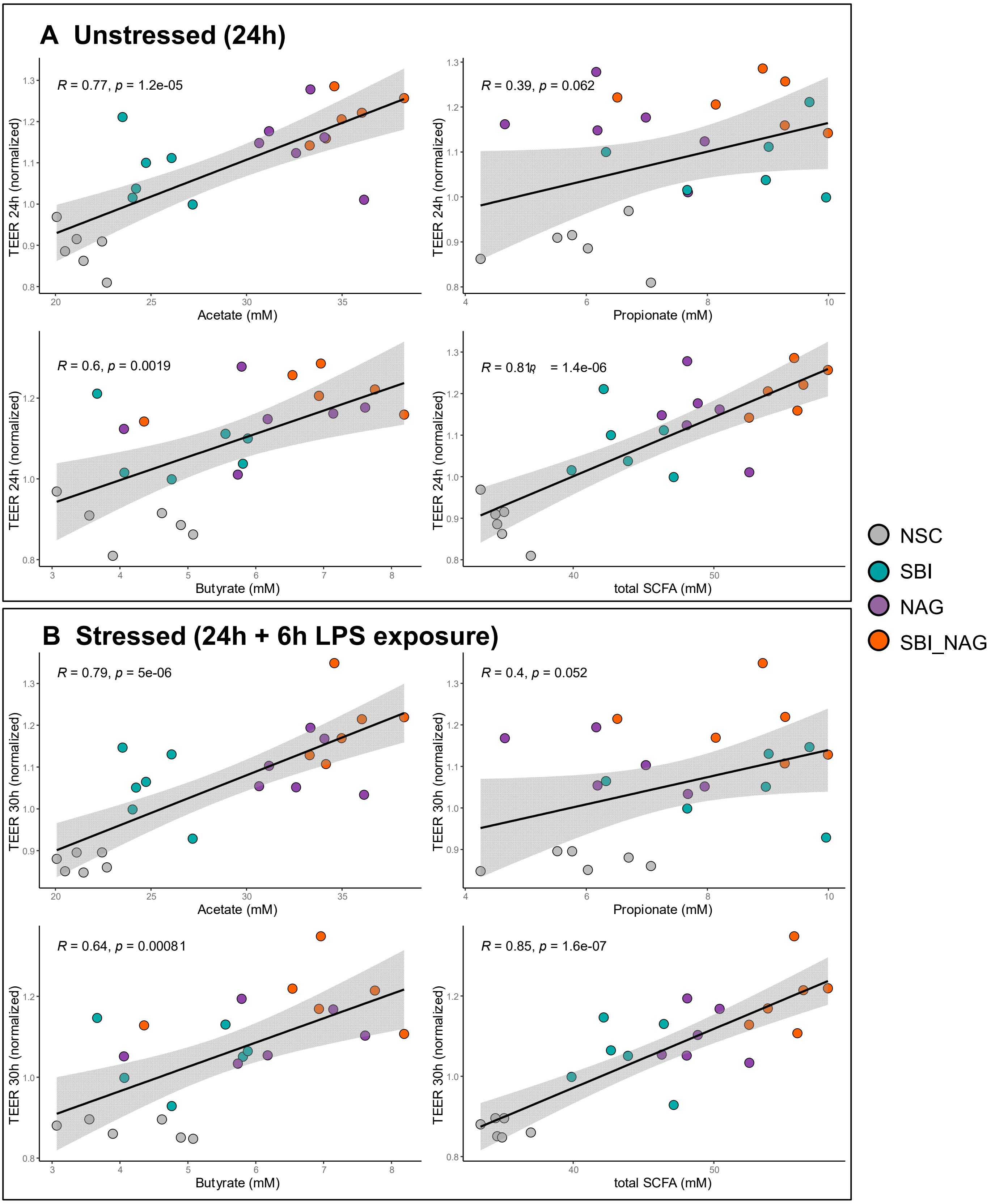

Figure S7. Gut barrier integrity (TEER) positively correlated with SCFA production. The correlation analysis was performed based on Spearman's rank correlation coefficient for TEER measured (**A**) at 24h incubation without LPS exposure and (**B**) after additional 6-hour LPS exposure (in total 30h). A *p*-value < 0.05 indicated a statistically significant correlation, while the *R*-value indicates the strength of the correlations (*R* < 0: negative correlation, *R* > 0: positive correlation).

nutrients

MDPI

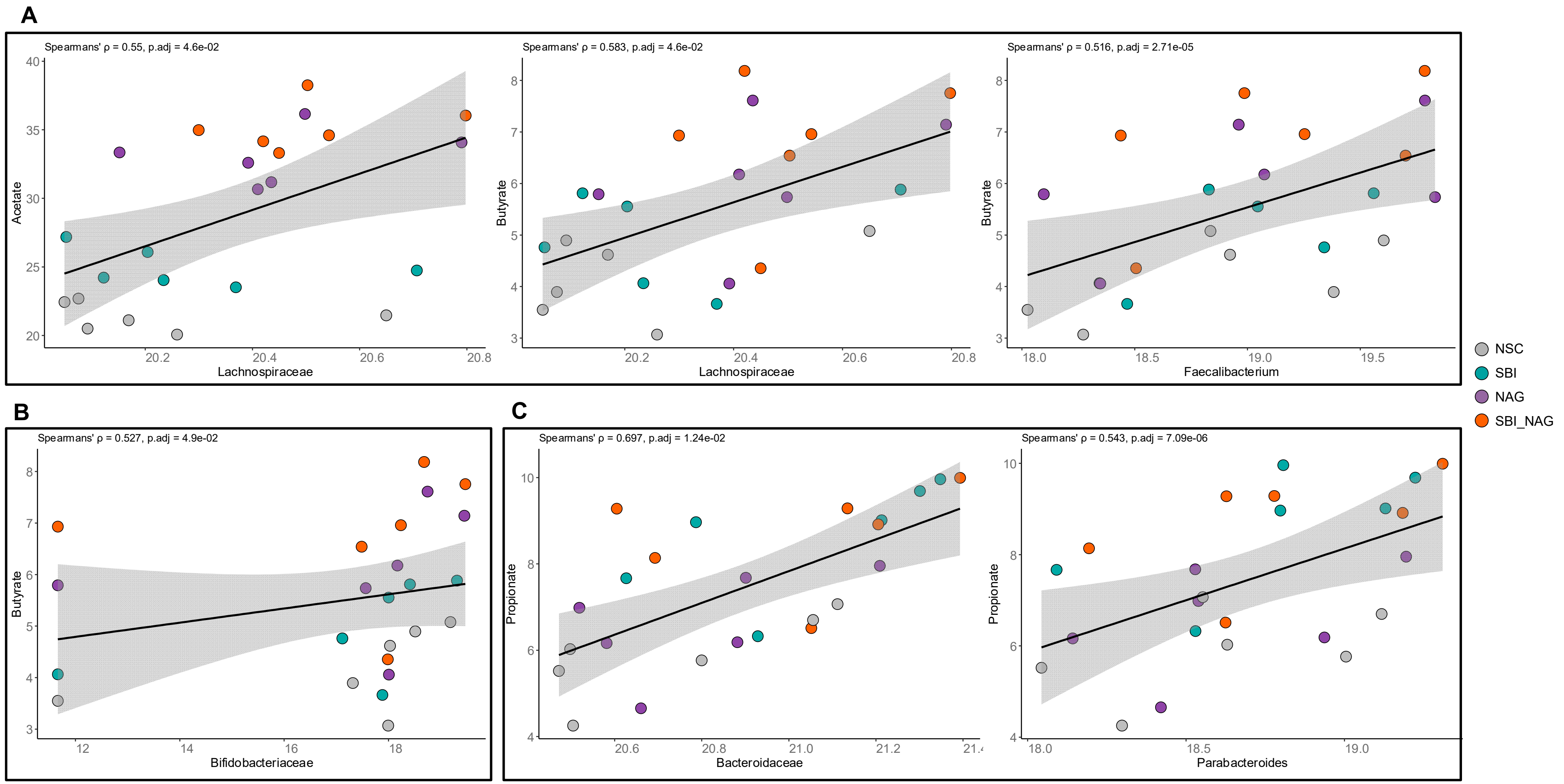

Figure S8. SCFA production correlated with specific taxa stimulated by SBI and/or NAG. The correlation analysis was performed based on Spearman's rank correlation coefficient. A *p*-value < 0.05 indicated a statistically significant correlation, while the *R*-value indicates the strength of the correlations (*R* < 0: negative correlation, *R* > 0: positive correlation). Significant positive correlations were noted between (**A**) *Lachnospiraceae* and *Faecalibacterium* (phylum Bacillota_A) and acetate and/or butyrate production, (**B**) *Bifidobacteriaceae* and butyrate production, and (**C**) *Bacteroidaceae* and *Parabacteroides* (phylum Bacteroidota) and propionate production.

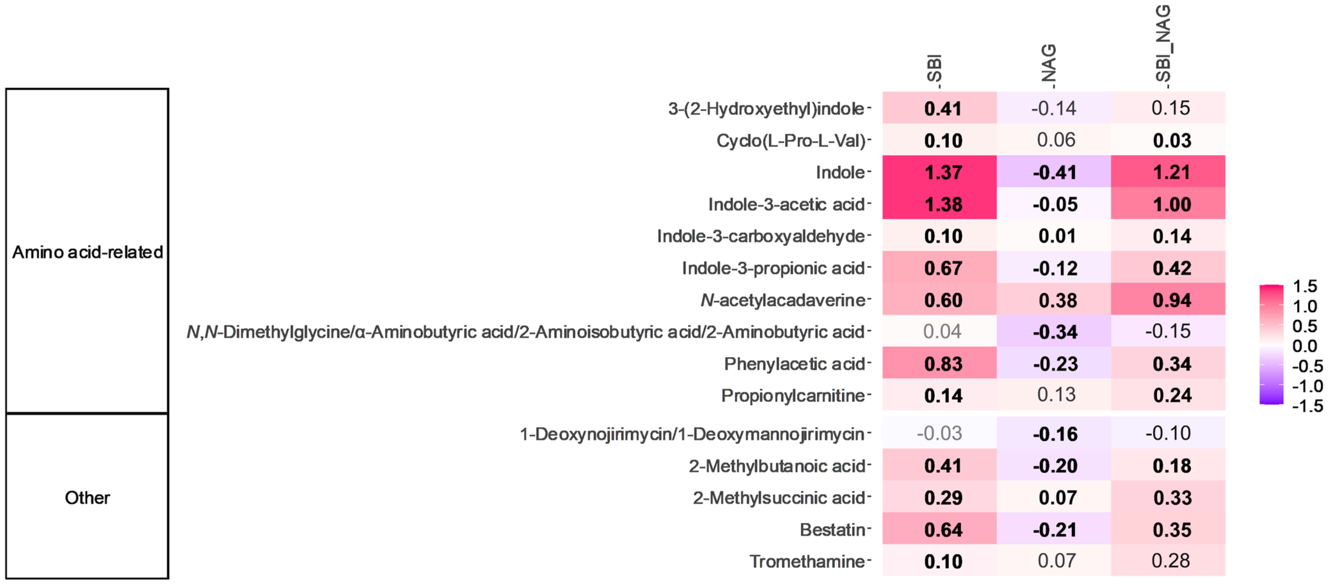

Figure S9. SBI, NAG and SBI_NAG affected specific metabolites. The heatmap displays significantly affected metabolites, expressed as $\log_2$ fold change in cell density (cells/mL) compared to the untreated control (NSC) at 24h of incubation in the SIFR® technology (n = 6). Bold numbers indicate significant treatment effects.

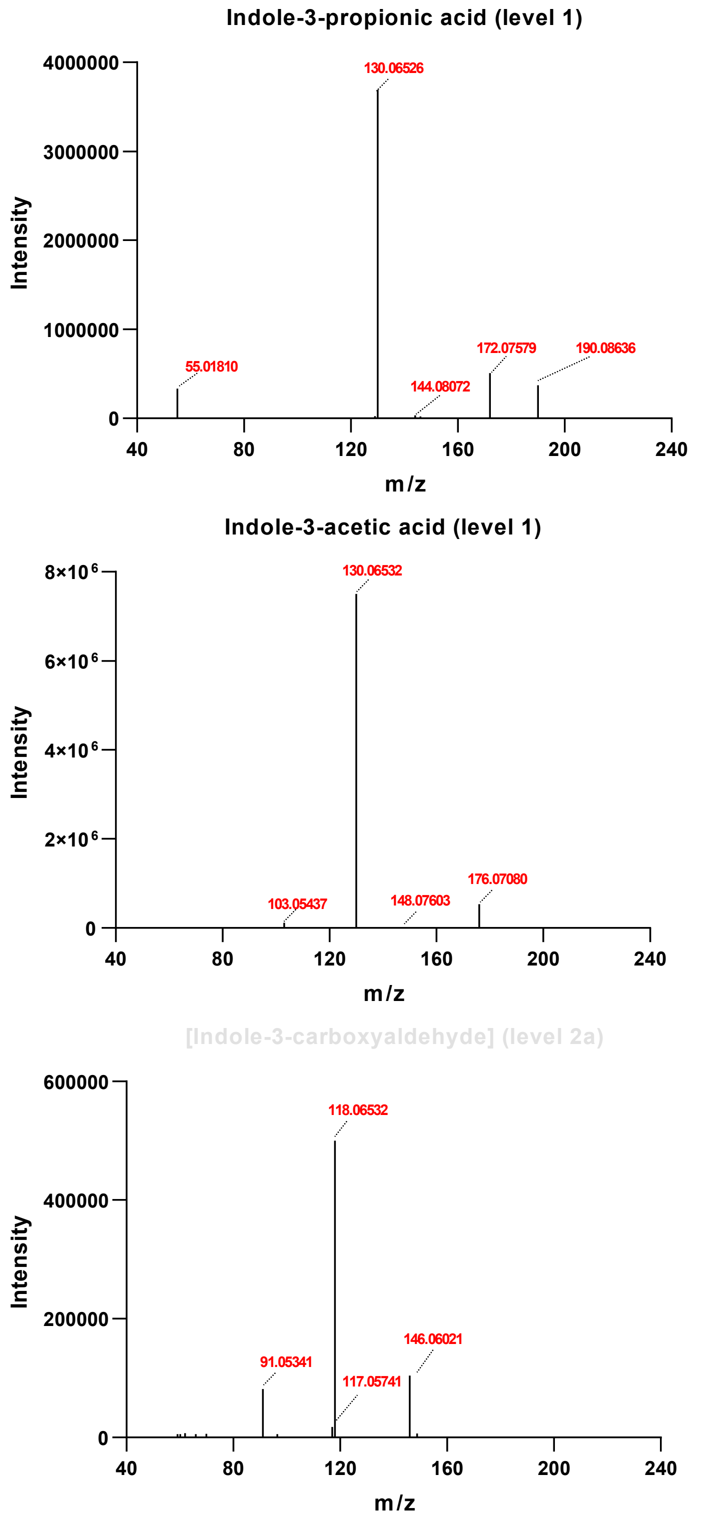

Figure S10. Representative MS/MS spectrum of the tryptophan derivative indole-3-propionic acid (IPA), indole-3-acetic acid (IAA) and indole-3-carboxyaldehyde (I3A). The level of identification is indicated for each compound, either level 1 (based on retention times (compared against in-house authentic standards), accurate mass (with an accepted deviation of 3 ppm), and MS/MS spectra) or level 2a (based on retention times (compared against in-house authentic standards), accurate mass (with an accepted deviation of 3 ppm)). m/z of characteristic fragment ions that matched with the library are indicated in red. As a note, for I3A (identified at level 2a), the MS/MS spectrum was visualized for the same precursor with nearly identical retention time. However, during compound identification, the recorded MS/MS spectra were not grouped with the distinct MS1 peak from the samples, leading to a level 2a classification.

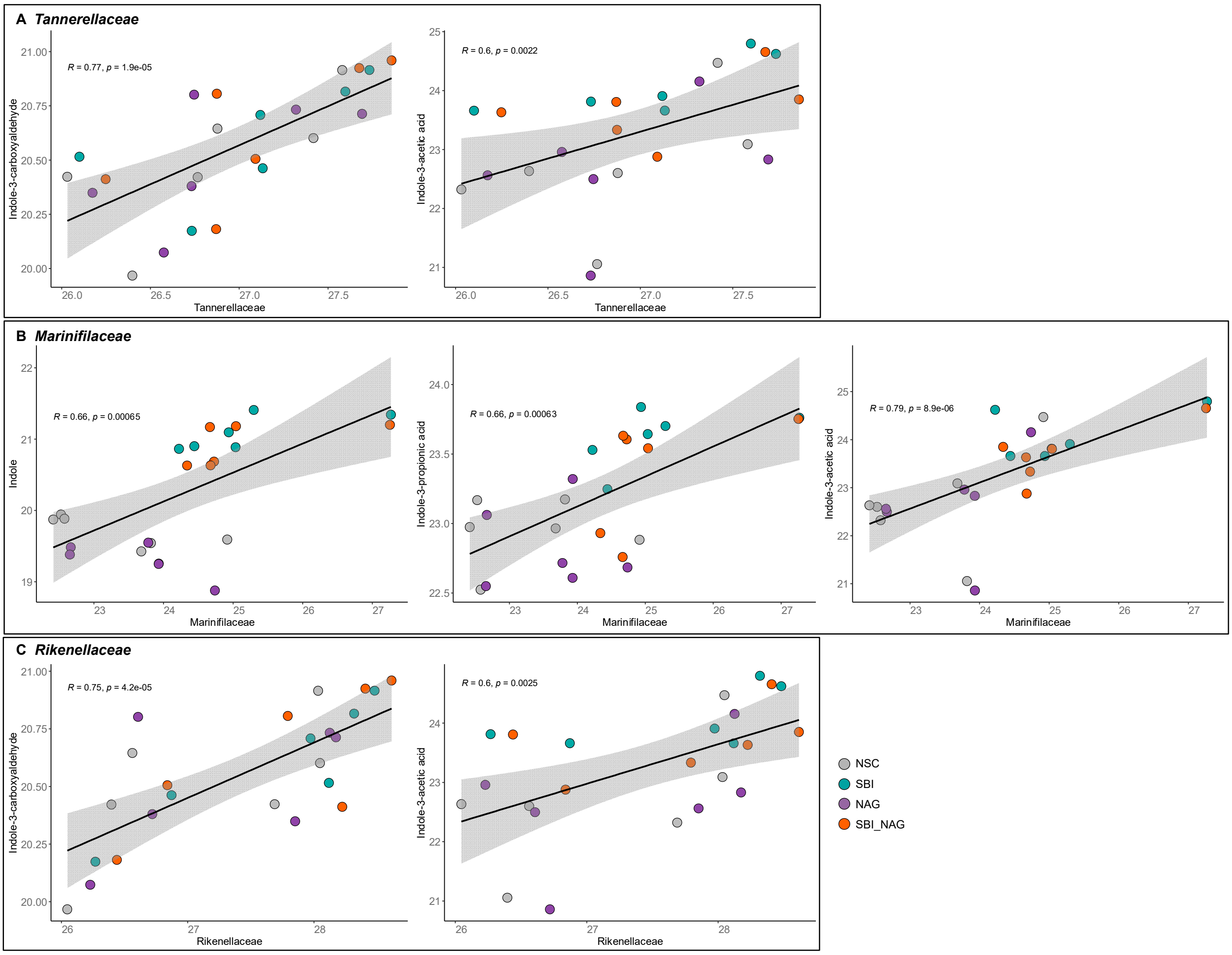

Figure S11. Bacteroidota families positively correlated with the production of various indoles. The correlation analysis was performed based on Spearman's rank correlation coefficient. A *p*-value < 0.05 indicated a statistically significant correlation, while the *R*-value indicates the strength of the correlations (*R* < 0: negative correlation, *R* > 0: positive correlation). Significant positive correlations were noted between different indoles and (**A**) *Tannerellaceae,* (**B**) *Marinifilaceae* and (**C**) *Rikenellaceae*.
